# The global prevalence of *Toxocara canis* among red foxes (*Vulpes vulpes*): A systematic review and meta-analysis

**DOI:** 10.1016/j.ijppaw.2024.100984

**Published:** 2024-09-04

**Authors:** Celia V. Holland, Zahra Geraili Afra, Soghra Valizadeh, Maryam Ebrahimi, Ali Rostami

**Affiliations:** a Department of Zoology, School of Natural Sciences, Trinity College Dublin, College Green, Dublin, 2, Ireland; b Social Determinants of Health Research Center, Health Research Institute, Babol University of Medical Sciences, Babol, Iran; c Department of Food Hygiene and Aquatic, Faculty of Veterinary Medicine, University of Tabriz, Tabriz, Iran; d Department of Parasitology and Mycology, School of Medicine, Shahid Beheshti University of Medical Sciences, Tehran, Iran; e Infectious Diseases and Tropical Medicine Research Center, Health Research Institute, Babol University of Medical Sciences, Babol, Iran

**Keywords:** *Toxocara canis*, Red foxes, Prevalence, Meta-analysis, Abundance

## Abstract

Red foxes play a crucial role in the life cycle and transmission of zoonotic pathogens, including *Toxocara canis*; however, comprehensive information on the prevalence of *T. canis* in red foxes (*Vulpes vulpes*) is lacking. In this meta-analysis we aimed to evaluate the global and regional prevalence of *T. canis* among red foxes. We searched PubMed, Scopus, and Google Scholar for studies reporting prevalence of *T. canis* in red foxes up to April 1, 2024. Using a random-effects model, we estimated pooled prevalences at global, regional, and national levels and assessed heterogeneity through subgroup and meta-regression analyses. The overall pooled global prevalence of *T. canis* infection in red foxes was 32.1% (95% CI, 28.5–35.6%), with the highest prevalence in Europe (34.6%, 30.9–38.3%) and the lowest in the Eastern Mediterranean (20.0%, 11.0–29.0%). In other regions, prevalences of *Toxocara* were as follows: Central Asia (33.1%, 26.8–39.4%), North America (23.6%, 10.6–36.6%), Western Pacific (21.3%, 5.2–37.4%), and Eastern Mediterranean & North Africa (20.0%, 11.0–29.0%). However, data from certain geographical regions are very limited (for example Greece, Austria, China and North Africa). Prevalence rates showed a decreasing trend over time. Subgroup analyses indicated higher prevalences in male red foxes (54.1%, 41.4–66.7%) compared to females (37.5%, 29.9–45.1%), and in juvenile red foxes (56.2%, 39.1–73.3%) compared to adults (33.4%, 23.2–43.6%). *T. canis* worm burdens were generally low, not exceeding an average of 4 worms per fox in most studies. Our findings reveal a substantial prevalence of *T. canis* infection in red fox populations worldwide (32.1%), highlighting their potentially significant role in perpetuating the transmission of infection to both companion animals and humans. Continued surveillance is essential to mitigate the risk of *Toxocara* transmission to companion animals and humans. However, a major remaining challenge is to assess the relative importance of the red fox as a contributor to environmental contamination with *Toxocara* ova. Further research is also needed to address study limitations and provide a complete global picture of *T. canis* epidemiology in red foxes and other wild animals, especially in underrepresented regions.

## Highlights

• This meta-analysis aimed to evaluate the global and regional prevalence of *T. canis* among red foxes.
• The overall pooled global prevalence of *T. canis* infection in red foxes was 32.1%.
• The highest prevalence was 34.6% - Europe; the lowest was 20.0% - E. Mediterranean, N. Africa.
• Abundances of *T. canis* were generally low, not exceeding an average of 4 worms per fox in most studies.

## Graphical abstract

**Figure:**
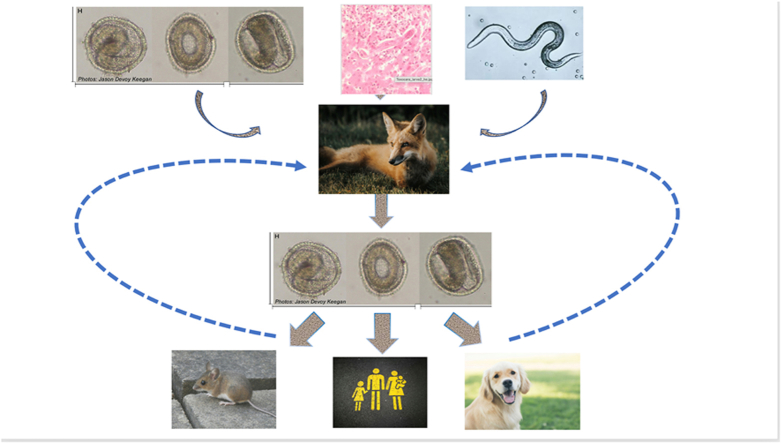

## 1 Introduction

*Toxocara* species are cosmopolitan parasitic nematodes that infect a wide range of companion, domestic and wild animals as definitive and paratenic hosts (Holland, 2017). Of the 26 recognized species of the genus *Toxocara,* only *Toxocara canis* and *Toxocara cati* are denoted to be zoonotic (Ziegler and Macpherson, 2019). Human infection occurs as a consequence of the accidental ingestion of eggs or larvae in contaminated soil, food or water (Rostami et al., 2019). As our knowledge increases, the public health significance of the disease known as toxocariasis, becomes more apparent with visceral, ocular, neurological and allergic manifestations typically associated with infection (Taghipour et al., 2021; Alizadeh Khatir et al., 2021; Darvish et al., 2021; Mohammadzadeh et al., 2020). In the first study of its kind, Antonopoulos et al. (2024) estimated that 91,714 DALYs per year are lost across all countries due to toxocariasis. Despite this increasing attention, diagnostic difficulties persist, particularly with respect to serology and neurotoxocariasis (Ma et al., 2018; Deshayes et al., 2016).

Transmission routes of *T. canis* are complex and involve the shedding of eggs into the environment from definitive hosts harbouring adult worms, the presence of larvae arrested in the tissues of domestic dogs, and in paratenic hosts that can be consumed as prey by definitive hosts or as food items by humans (Holland, 2017). More recently, *Toxocara* eggs have been detected on the hair of domestic dogs and foxes, although the evidence to date suggests that if dogs are well cared for, egg embryonation is unlikely (Keegan and Holland, 2012).

The significance of wild carnivores as definitive hosts of *T*. *canis* and *T*. *cati* has received much less attention compared to that given to domestic dogs (Rostami et al., 2020a) and cats (Rostami et al., 2020b). In a recent review, the wild canids that have received most attention in the published literature include the red fox (*Vulpes vulpes*), the wolf (*Canis lupus*) and the golden jackal (*Canis aureus*) (Holland, 2023). However, red foxes would appear to be the most significant wild species in terms of their potential to transmit *Toxocara* to domestic dogs and humans via environmental contamination. This can be explained by their increasing population densities, encroachment into urban areas and their dietary preferences for a wide range of potential paratenic hosts (Holland, 2023). The red fox (*Vulpes vulpes*) is one of the most widespread wild canids, with a distribution spanning the entire Northern Hemisphere from the Arctic circle to southern North America, Europe, North Africa, the Asiatic steppes, India and Japan (Hoffmann and Sillero-Zubiri, 2016). With a global population estimated at over 10 million individuals, red foxes play a significant role in the ecology of various ecosystems (World Population Review). Their adaptability to diverse habitats, including urban areas, has led to a significant increase in their population density in many regions (Waindock et al., 2021).

Therefore, the main aim of this study was to systematically review and conduct a meta-analysis of the global prevalence of *T. canis* among red foxes. This will provide a comprehensive understanding of the epidemiological patterns and potential risks associated with this zoonotic parasite in an important widespread wild host.

## 2 Methods

This comprehensive systematic review and analysis adhered to the established guidelines for systematic reviews and meta-analyses, including the Preferred Reporting Items for Systematic Reviews and Meta-Analyses (PRISMA) (Moher et al., 2015) and Meta-Analysis of Observational Studies in Epidemiology (MOOSE) (Stroup et al., 2000) frameworks, as outlined previously.

### 2.1 Search strategy and study selection

To identify studies eligible for meta-analysis, we searched PubMed and Scopus from their inception to April 1, 2024. We conducted a comprehensive search using combinations of the following keywords with the Boolean operators “OR” and “AND”: “*Toxocara*,” “*Toxocara canis*,” “toxocariasis,” “*Toxocara* spp.,” “prevalence,” “occurrence,” “red fox,” “fox,” and “*Vulpes vulpes*.” The search strategies for PubMed and Scopus are detailed in Fig. S1. To retrieve additional relevant studies and gray literature, we performed a search in Google Scholar (first 40 pages) and manually searched the reference lists of retrieved citations and published reviews. No language or geographic limitations were applied, but we examined only the abstracts, in studies published in languages other than English. All retrieved articles were imported into EndNote software, and duplicates were removed manually. The titles and abstracts were assessed by one author (C.V.H.), and irrelevant studies were eliminated. The remaining studies were evaluated for inclusion based on the following criteria: (i) Original research studies with a cross-sectional design reporting the prevalence of *Toxocara* in red foxes (*Vulpes vulpes*); (ii) Sample size of >20 red foxes; (iii) Use of faecal examination to detect *Toxocara* eggs or postmortem examination to identify *Toxocara* worms. Studies were excluded if they had no precise information on sample size or prevalence rates, involved experimentally infected animals, focused on fox species other than *Vulpes vulpes*, or were diagnostic studies, or lacked original data (e.g., reviews, systematic reviews *etc*.).

### 2.2 Data extraction and study quality assessment

Two independent experts (C.V.H. and A.R.) extracted information from the eligible studies, using a proforma Microsoft Excel spreadsheet (2016 version, Microsoft, Redmond, WA, USA) and any inconsistencies were discussed to reach a consensus. The following information was collected for each study: the first author's last name, publication year, country, city or area, start and end dates of the sampling, diagnostic method, types of samples tested (worms or eggs), geographical location (rural or urban area), and age and sex of red foxes (if available), sample size, number of *Toxocara canis*-positives samples, prevalence, and associated 95% confidence interval (CI). All geographical areas (i.e., countries and cities) studied were classified into different continental regions according to WHO-defined regions, with slight modifications based on geographic, economic, and climatic differences, as well as the number of available studies (Europe, Central Asia, Eastern Mediterranean & North Africa, North America, and Western Pacific). Since there were available studies for most countries in Europe, we divided Europe into four sub-regions (Northern, Southern, Western, and Eastern Europe) to provide more detailed estimates and interpretations. To evaluate the quality and risk of bias for each eligible publication, we utilized the Joanna Briggs Institute (JBI) Prevalence Critical Appraisal Tool (Munn et al., 2014). The risk of bias was evaluated by calculating the percentage of 'yes' responses to ten specific questions. These questions pertained to aspects such as obtaining a representative sample, appropriately recruiting subjects (foxes), determining sample size, describing the setting and subjects, measuring outcomes, ensuring measurement reliability, analyzing data, examining subpopulations, and adjusting for confounding factors for each study. Two trained authors (A.R. and M.E.) independently evaluated the quality of each record, and any discrepancies were resolved by discussion. Publications were ranked as having "low", "moderate", or "high" risks of bias if they received scores of 7–10, 4–6, or 1–3, respectively. Due to insufficient and/or heterogenic information in available studies, data on abundance and intensity of *T. canis* were not subjected to the meta-analysis but rather are given as descriptive statistics for any relevant study.

### 2.3 Data synthesis and statistical analysis

All statistical analyses were conducted using STATA version 13 (STATA Corp., College Station, Texas). To pool the prevalence estimates from the raw cell counts, we employed a Freeman-Tukey double arcsine transformation and calculated 95% confidence intervals for the individual studies (Doi and Xu, 2021). In this meta-analysis, we utilized random-effect models as described by DerSimonian and Laird (2015) to generate pooled global, regional, and national prevalence estimates with 95% confidence intervals, using the metaprop command in Stata software. Between-study heterogeneity was assessed using Cochran's Q and the *I*^2^ statistic, with *I*^2^ values of 50% or more considered substantial (Higgins et al., 2003). To explore the sources of heterogeneity, we performed meta-regression analyses and subgroup analyses. Meta-regression was used to examine the effect of time on the prevalence of *T. canis*. In the subgroup analyses, we estimated the prevalence of *T. canis* in red foxes across different geographic regions, publication years, sampling dates, age groups (juvenile and adult foxes), sexes (males and females), residency status (urban and rural), types of tested samples, and diagnostic methods. We did not undertake a formal assessment of publication bias, mainly considering the recognized limitations of common evaluation methods, such as Egger's tests and standard funnel plots, when applied to proportional meta-analyses. Specifically, there are two key reasons underlying this decision. First, the suitability of these statistical tools for accurately detecting publication bias in the context of proportional data remains unclear. Second, it is important to recognize that detection techniques for publication bias were originally developed with the assumption that studies with favorable results are more likely to be published - an assumption that does not apply as clearly to meta-analyses focused on proportional outcomes, where the distinction between "favorable" and "unfavorable" results is less well-defined (Barker et al., 2021; Hunter et al., 2014). The significance level was set at a p-value of less than 0.05.

## 3 Results

### 3.1 Study characteristics

**Fig. 1:**
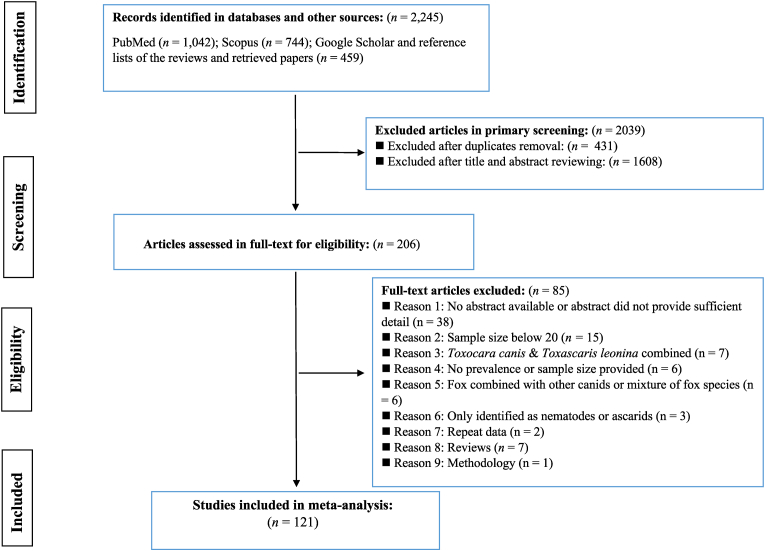
Flow chart of the study selection process showing inclusion and exclusion criteria for the identified studies.

Fig. 1 summarizes the study selection process and the number of studies at each stage. Out of 2245 articles retrieved, 2039 were excluded after removing duplicates and screening titles and abstracts. After applying the inclusion and exclusion criteria, 121 unique studies (128 datasets, Table S1) comprising 32,580 red foxes from 40 countries were eligible for the meta-analysis. These datasets represented five geographical regions: 100 from Europe (27,795 samples), 10 from North America (1189 samples), 10 from the Eastern Mediterranean and North Africa (856 samples), six from the Western Pacific (2529 samples), and two from Central Asia (213 samples). The included studies were published between 1943 and 2024, with a median of 254 red foxes per study (range: 20 to 3138). A total of 101 studies used postmortem examination to detect worms, while 27 used fecal examination to find *T. canis* eggs. The raw prevalence of *T. canis* among red foxes in individual studies ranged from 0.0% to 69.5%. The main characteristics of the included studies are presented in Table S1.

### 3.2 Results of meta-analysis

**Fig. 2:**
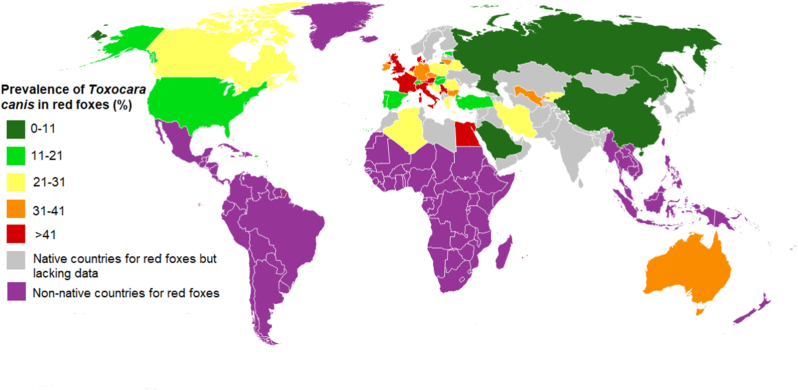
Global prevalence of *Toxocara canis* in red foxes. (For interpretation of the references to colour in this figure legend, the reader is referred to the Web version of this article.)

As depicted in Table 1, 10,828 of 32,580 red foxes were diagnosed as infected with *T. canis*, resulting in an overall, pooled global prevalence of 32.1% (95% CI, 28.5–35.6%) (Table 1), with substantial heterogeneity among studies (*I*^2^ = 98.84%, P < 0.001). In relation to geographical regions, prevalences were (in descending order, with the 95% CIs): 34.6% (30.9–38.3%) in Europe; 33.1% (26.8–39.4%) in Central Asia; 23.6% (10.6–36.6%) in North America, 21.3% (5.2–37.4%) in the Western Pacific; and 20.0% (11.0–29.0%) in the Eastern Mediterranean & North Africa region. With regards to European sub-regions prevalences were 50.6% (43.7–57.5%) in Northern Europe, 41.6% (34.8–48.4%) in Western Europe, 29.6% (23.2–36.0%) in Southern Europe, and 23.4% (18.3–28.5%) in Eastern Europe. A map produced using geographic information system (GIS), summarizing the prevalence of *T. canis* among red foxes in different countries, is shown in Fig. 2. For countries with two or more eligible datasets, the Netherlands (66.2%), Denmark (60.7%), Great Britain (56.7%), Italy (48.4%), France (41.6%), Germany (40.1%), Ireland (38.9%), and Bulgaria (37.8%), all in Europe, and Australia (31.4%) in the western Pacific region, exhibited some of the highest prevalence estimates. Additional details pertaining to the prevalence of *T. canis* infection in red foxes in the individual countries are presented in Table 1 and Fig. 2.

**Table 1:** Global, and regional pooled prevalence of *T. canis* infection in red foxes (*Vulpes vulpes*).

Regions/country^a^	Number datasets	Number of red foxes screened	Number of foxes positive for *T. canis* infection	Pooled prevalence % (95% CI)
**Global**	**128**	**32,580**	**10,828**	**32.1 (28.5**–**35.6)**
**Europe**	**100**	**27795**	**9420**	**34.6 (30.9**–**38.3)**
**Northern Europe**	**19**	**4486**	**2415**	**50.6 (43.7**–**57.5)**
Denmark	5	1563	934	60.7 (55.3**–**66.2)
Great Britain	8	2160	1223	56.7 (51.4**–**61.9)
Ireland	3	255	99	38.9 (13.8**–**64.0)
Lithuania	1	269	109	40.5 (34.8**–**46.5)
Estonia	2	239	50	18.8 (13.9**–**23.7)
**Western Europe**	**21**	**10026**	**3248**	**41.6 (34.8**–**48.4)**
Netherlands	3	344	228	66.2 (58.5**–**73.9)
France	3	373	165	41.6 (29.0**–**54.3)
Germany	10	7088	2401	40.2 (33.1**–**47.2)
Austria	1	233	100	42.9 (36.7**–**49.3)
Belgium	2	279	74	24.8 (19.8**–**29.7)
Switzerland	2	1709	280	14.1 (12.5**–**15.7)
**Southern Europe**	**35**	**5550**	**1682**	**29.6 (23.2**–**36.0)**
Italy	10	1748	836	48.4 (35.3**–**61.6)
Greece	1	314	90	28.7 (23.9**–**33.9)
Andorra	1	53	11	20.7 (12.0**–**33.5)
Portugal	6	501	80	19.4 (9.5**–**29.4)
Spain	11	1690	269	16.2 (9.6**–**22.8)
Slovenia	1	428	164	38.3 (33.8**–**43.0)
Croatia	1	85	24	28.2 (19.8**–**38.6)
Serbia	**2**	**395**	**122**	26.4 (22.3**–**30.5)
Bosnia & Herzegovina	2	334	85	25.2 (20.5**–**29.8)
**Eastern Europe**	**25**	**7733**	**2075**	**23.4 (18.3**–**28.5)**
Bulgaria	2	356	144	37.8 (33.0**–**42.7)
Poland	11	4173	1205	23.6 (18.3**–**28.9)
Yugoslavia	1	459	231	50.3 (45.8**–**54.9)
Czech Republic	1	40	15	37.5 (24.2**–**53.0)
Belarus	1	94	24	25.5 (17.8**–**35.2)
Romania	4	673	190	21.43 (5.57**–**37.30)
Slovak Republic	3	1810	253	15.1 (7.6**–**22.7)
Hungary	1	100	12	12.0 (7.0**–**19.8)
Russia	1	28	1	3.6 (0.6-17**–**7)
**Central Asia**	**2**	**213**	**71**	**33.1 (26.8**–**39.4)**
Uzbekistan	1	62	25	40.3 (29.0**–**52.7)
Kyrgyzstan	1	151	46	30.5 (23.7**–**38.2)
**North America**	**10**	**1189**	**235**	**23.6 (10.6**–**36.6)**
Canada	4	487	128	28.7 (6.9**–**50.4)
United States	6	702	107	20.4 (2.9**–**37.9)
**Western Pacific**	**6**	**2529**	**929**	**21.3 (5.2**–**37.4)**
Australia	4	2464	929	31.5 (20.9**–**42.0)
China	2	65	0	1.3 (0.1**–**11.2)
**Eastern Mediterranean & North Africa**	**10**	**856**	**174**	**20.0 (11.0**–**29.0)**
Egypt	1	41	20	48.8 (34.2**–**63.5)
Algeria	1	22	5	22.7 (10.1**–**43.4)
Iran	4	2023	59	21.1 (3.5**–**38.6)
Turkey	3	532	90	16.8 (13.7**–**20.0)
Saudia Arabia	1	58	0	0.85 (0.1**–**7.6)

a Regions and countries are sorted based on prevalence rates.

**Fig. 3:**
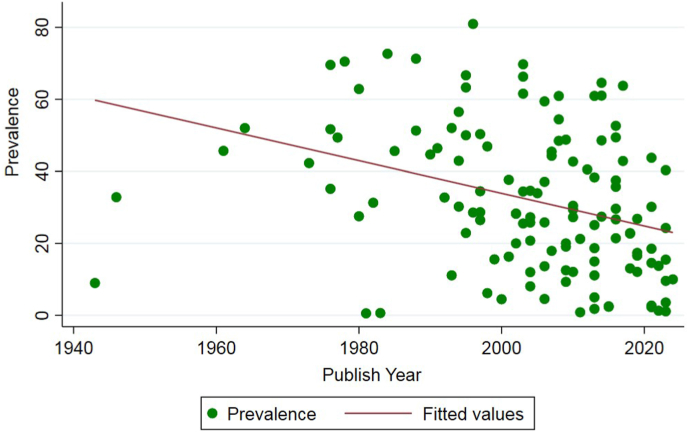
Random-effects meta-regression analyses of the prevalence of *Toxocara canis* infection in red foxes according to publication year, showing a statistically significant downward trend in prevalence in recent years. Values on the y axis are prevalence rates. (For interpretation of the references to colour in this figure legend, the reader is referred to the Web version of this article.)

In all subgroup analyses that considered year of publication, the highest prevalence rates were estimated for studies published or implemented before 1990 and lowest prevalences were estimated for studies published in 2020–2024 or implemented between 2010 and 2019 (see Table 2). In addition, meta-regression analyses for the year of publication (coefficient [*C*] = −0.004, P < 0.001; Fig. 3) and the year the data collection started (*C* = −0.003, P = 0.002; Fig. S2) and the year data collection ended (*C* = −0.003, P = 0.004; Fig. S3) showed significant decreasing trends in prevalence over time.

**Table 2:** Prevalence of *T. canis* infection in red foxes (*Vulpes vulpes*) based on sub-group analyses.

Variable/subgroups	Number studies	Number of red foxes screened	Number of foxes positive for *T. canis* infection	Pooled prevalence (95% CI)
**Year of publication**				
Before 1990	20	7847	2973	45.6 (34.3**–**56.9)
1990–1999	21	6605	2424	39.2 (32.2**–**46.1)
2000–2009	34	9309	3245	31.6 (25.0**–**38.2)
2010–2019	38	6872	1769	27.9 (22.5**–**33.2)
2020–2024	15	1947	417	14.9 (8.6**–**21.1)
**Start date of sampling**				
Before 1990	30	11,029	4039	40.3 (31.7**–**48.8)
1990–1999	19	8001	3408	42.7 (34.9**–**50.4)
2000–2009	27	6657	1826	31.6 (26.1**–**37.2)
2010–2021	30	4089	818	21.5 (16.7**–**26.4)
**End date of sampling**				
Before 1990	21	8752	3124	32.1 (28.5**–**35.6)
1990–1999	17	4946	1904	42.4 (33.5**–**51.2)
2000–2009	32	9896	3491	34.4 (27.9**–**40.8)
2010–2022	35	6121	1529	23.1 (18.5**–**29.0)
**Egg count/worm count**				
Worm count	101	27,466	10,028	32.1 (31.9**–**40.4)
Egg count	27	5114	800	15.5 (11.8**–**19.3)
**Diagnostic method**				
Flotation (egg counts)	22	4946	780	16.4 (12.3**–**20.5)
Sedimentation & counting technique (worm counts)	17	3605	1509	38.4 (30.3**–**46.5)
Intestinal scraping (worm counts)	21	6091	2082	30.5 (23.6**–**37.6)
Other^a^	27	7622	2875	35.9 (27.1**–**44.6)
Method not reported	41	10,316	3582	35.8 (29.2**–**42.4)
**Age**				
Juvenile	14	1597	872	56.2 (39.1–73.3)
Adult	14	2805	1052	33.4 (23.2–43.6)
**Sex**				
Male	21	3431	1734	54.1 (41.4–66.7)
Female	21	2777	1027	37.5 (29.9–45.1)
**Urbanization**				
Urban	8	943	428	45.6 (32.5–58.6)
Rural	4	719	292	43.1 (10.1–76.1)
**Risk of bias**				
Low risk	72	29543	9869	33.8 (29.4–38.2)
Moderate risk	30	2226	733	33.0 (23.7–42.3)
High risk	26	811	226	25.4 (18.5–32.4)

a Visual inspection (worms), washing & sieving (worms), scraping, filtration and counting technique (SFCT)(worms), mini FLOTAC (eggs), McMaster technique (eggs), metabarcoding, DNA extraction and PCR.

In subgroup analyses, the pooled prevalence of *T. canis* infection in male and female red foxes was 54.1% (41.4–66.7%) and 37.5% (29.9–45.1%), respectively (P < 0.001). The pooled prevalence of *T. canis* infection in juvenile red foxes 56.2% (39.1–73.3%) was higher than adults 33.4 (23.2–43.6%) (P < 0.001). Moreover, the prevalence of *T. canis* infection in red foxes from urban areas (45.6%, 32.5–58.6%) was slightly higher than in red foxes from rural areas (43.1%, 10.1–76.1%), but the difference was not statistically significant (P = 0.62). All these data sets were from Europe except one Australian city. In subgroup analyses, according to type of tested samples, the pooled prevalence of *T. canis* infection was higher in studies that performed postmortem examination to identify *Toxocara* worms (32.1%, 31.9–40.4%) than in those that performed faecal examination to detect *Toxocara* eggs (15.5%, 11.8–19.3%; P < 0.001). With respect to risk of bias, studies with low, moderate and high risk of bias demonstrated prevalences of 33.8% (29.4–38.2%), 33.0% (23.7–42.3), and 25.4% (18.5–32.7%), respectively. More details on subgroup analyses are presented in Table 2.

### 3.3 Measures of worm burden in red foxes

Data on the worm burdens of *T. canis* in red foxes are sparse in the literature. However, the data that are available provide important information and in general, indicate similar patterns of infection across regions. Two terms are used to describe the worm burden of *T. canis* in red foxes. As defined by Bush et al. (1992), mean abundance represents the mean worm burden including both infected and uninfected hosts (zeroes) whereas mean intensity respresents the mean worm burden that includes only infected hosts. This explains why mean abundance values are always lower than those of mean intensity. Out of the total of 121 studies, 16.52% provided data on mean abundance with modest values ranging from a mean of 0.3–17.1 (Table S2). In fact, abundances did not exceed 6 worms per fox with the exception of the Danish study by Willingham et al. (1996), in which a mean of 17.1 was recorded for a relatively low sample size of 21 red foxes. Similarly, intensity values were reported from 21.5% of the studies, with values ranging from a mean of 1.4–20.4 (Table S2). Intensity did not exceed 12 worms per fox with the exception of a mean of 20.4 in Austrian foxes described by Suchentrunk and Sattmann (1994). Worm burden ranges showed more variation with values as low as 1–9 and highest at 1–213 (Table S2).

Measures of aggregation were even less frequently available with only seven studies (5.46%) recording either k values or variance to mean ratios. Aggregation is a significant and unbiquitous epidemiological papttern observed in macroparasite populations. Put simply, most hosts carry few or no parasites and a small proportion of hosts carry heavy infections (Holland, 2009). The value of k is an inverse measure of aggregation and values less than 1 indicate aggregation. In contrast, variance to mean ratios that are greater than 1 represent aggregation (Shaw and Dobson, 1995). K values were 0.10 (Magi et al., 2016), 0.233 (Saeed et al., 2006), 0.253 (Di Cerbo et al., 2008), 0.288 (Richards et al., 1995) and 0.30 (Ziadinov et al., 2010). Variance to mean ratios were 13.10 (Fiocchi et al., 2016), 13.3 (Miljevic et al., 2019), 19 (Saeed et al., 2006) and 20.45 (Richards et al., 1995). Only two studies provided both k values and variance to mean ratios (Richards et al., 1995; Saeed et al., 2006). All these values provide evidence of aggregation.

## 4 Discussion

In this meta-analysis, based on 121 studies from 40 countries and a population of over 32,000 foxes, we have reported an overall pooled global prevalence of *T. canis* infection of 32.1% in red foxes. Western Europe has the highest prevalence at 39.4%, and the lowest prevalence at 20.0% was recorded for the Eastern Mediterranean. In comparison, in a meta-analysis of *T. canis* in dogs derived from 60 countries and a population of 13, 010, 004 dogs, a lower overall prevalence of 11.1% was reported with the highest prevalence of 19.2% from the Eastern Mediterranean and the lowest from the Western Pacific (6.4%)(Rostami et al., 2020a). European dogs had a prevalence of 10.8%. Rostami and colleagues also disaggregated their analysis to report the global prevalence of *T. canis* in stray dogs and this was 18.8%. Of particular note is the very low prevalence of *T. canis* in dogs sampled from Australia (3.7%) where control of *T. canis* is more successful than in other parts of the world (Thompson, 2023). This contrasts with a prevalence of 34.5% in Australian red foxes derived from 4 studies (Table 1) and highlights the potential of infection in foxes to spill over into both domestic animals and humans (Bezerra-Santos et al., 2023). Therefore, based on the available data, the prevalence of *T. canis* in red foxes is higher than that of domestic dogs, including stray dogs.

These findings should also be placed in the context of the significant increases in fox density, observed particularly in parts of Europe but also in Australia, where red foxes are an introduced species. Such increases can be attributed to a number of factors including successful anti-rabies vaccination, reduced culling of foxes, their adaptation to new habitats such as suburban or urban areas and the availability of abundant, often anthropogenic food sources (Gloor et al., 2001; Contesse et al., 2004; Goszczynki et al., 2008; Waindock et al., 2021).

Studies on the prevalence of *T. canis* have been conducted over a long period of time from as early as 1938 up to the present with the number of studies performed increasing over time (Table S1). However, the prevalence of *T. canis* in red foxes, based on the available data, demonstrates a decrease over time. Explanations for this may relate to foxes feeding more regularly on anthropogenic food sources rather than potential paratenic hosts or reduced environmental contamination from enhanced treatment of domestic dogs. However, this trend may not reflect a biological reality but rather inherent study and sample size biases.

As reported here, the average worm burdens of *T. canis* in red foxes are low, rarely exceed 4 worms and the majority of studies report fewer than 5 worms per fox (Table S2). In general, data on the abundance of *T. canis* infection in dogs is available as egg counts derived from diagnostic testing of faecal samples, in contrast to foxes where most of the data is from worm counts at necroscopy (Morgan et al., 2013). Some limited data on comparative worm burdens is available from stray dogs and rural foxes sampled in Ireland (Roddie et. al. 2008a, 2008b). The prevalence of *T. canis* was higher in foxes than stray dogs (61% versus 39%) mirroring what has been described here. Furthermore, worm burdens from this small sample of Irish foxes are similar to those from the present study (4 ± S.D. 8). In contrast, the worm burdens of stray dogs are considerably higher (29.13 ± S.D.7.63). However, the relatively small sample sizes, the stray status of the dogs sampled, the inclusion of puppies and the local context, mean that this comparison should be interpreted with caution.

The majority of studies that contributed to the pooled prevalence estimates were based upon an analysis of worm burdens at postmortem (n = 101) versus egg counts (n = 27) and the pooled prevalence of *T. canis* infection was higher in studies that performed postmortem examination to identify *Toxocara* worms (32.1%) than those that performed faecal examination to detect *Toxocara* eggs (15.5%). In support of these observations, several studies have compared the sensitivity of detection using egg counts versus worm counts to measure prevalence of *T. canis* in the same sample of foxes. Not surprisingly, given the inherent variability in egg detection (Byrne et al., 2018), such studies have demonstrated the lower efficacy of egg counts as a measure of prevalence. For example, Martinez-Carrasco et al. (2007) recorded prevalences of 12.5% by egg count versus 45.5% by worm count in Spanish red foxes and Saeed and Kapel (2006) recorded the prevalence based on eggs as 41%, but that based on worms as 76% in Danish foxes. In a recent comprehensive study, Marchiori et al. (2023) compared copromiscroscopy utilising a classical flotation technique (FT) to a scraping filtration and counting technique (SFCT) in 150 red foxes from Poland. Both *T*. *canis* and *Toxascaris leonina* were combined as ascarids in their analysis. Concordance values were 62.7% and sensitivity was 36.3% but the latter increased to 46.1% after the exclusion of single-sex infections and infections solely by immature worms.

Pooled prevalences of *T. canis* in juvenile versus adult foxes revealed higher prevalences in juveniles compared to adults. This finding is consistent with the patterns observed in domestic dogs (Mohamed et al., 2009; Rostami et al., 2020a) and its explanation lies in the efficiency of vertical transmission to puppies compared to the more environmental routes by which adult foxes may become infected, e.g. via contamination with eggs or consumption of paratenic hosts (Schnieder et al., 2011; Otranto and Deplazes, 2019). Male foxes have higher pooled prevalences compared to female foxes. These trends are also found in detailed individual studies on the epidemiology of *T. canis* in red foxes. For example, Richards et al. (1993) recorded higher prevalences of *T*. *canis* among male versus female red foxes and juvenile versus adult red foxes in the UK. In Denmark, Saeed et al. (2006) found that the prevalence and abundance of *T*. *canis* was higher in male versus females red foxes and in cubs versus older animals. In a very detailed study that included an analysis of worm fecundity, Saeed and Kapel (2006) revealed that female *Toxocara* worm fecundity was lower in female compared to male red foxes and higher in cubs versus young and adult foxes. In general, male hosts tend to have higher helminth parasite burdens compared to female hosts (Oliver-Guimerá et al., 2017) and one possible explanation for this is the immunosuppressive effect of testosterone (Curi et al., 2017).

Given the relatively high prevalences observed for *T. canis* in red foxes, we need to consider the implications both in terms of environmental contamination and potential transmissibility to domestic dogs and humans. The red fox has the broadest geographical range of any species in the order Carnivora, being widely distributed across the entire Northern Hemisphere from the Arctic circle to southern North America, Europe, North Africa, the Asiatic steppes, India and Japan (Hoffmann and Sillero-Zubiri, 2016). In many European countries, red fox density has increased and this can be attributed to successful rabies vaccination campaigns, an increase in anthropogenic food sources and environmental factors (Gloor et al., 2001). As stated by Bateman and Fleming (2012) “anthropogenically altered landscapes vary along a gradient from rural farmlands through to the centres of cities, the ultimate urban habitat”. In their elegant figure, they compare how a variety of wild carnivores inhabit different habitats and the resources and impediments therein. The habitats include undeveloped landscape, rural farmland, villages and small towns, city suburbs and city centres. The relative length and direction of the arrows depicted demonstrate how red foxes are present throughout all categories, extending to cities across the world (Bateman and Fleming, 2012).

One explanation for the success of red foxes is their dietary plasticity (Castaneda et al., 2022). In a comprehensive analysis, Castaneda and colleagues studied the diet of red foxes at 276 locations in five continents. Despite substantial variation across its range, small mammals and invertebrates were the most frequently recorded dietary items. However, these authors emphasised the ability of red foxes to exploit novel anthropogenic food resources as key to their success in highly anthropogenic habitats. Soe et al. (2017) conducted a Europe-wide analysis of red fox diet, using data from 66 studies in 17 European countries. The composition of red fox diet was found to vary considerably in response to latitude, human influence, and warm and cold periods. Dietary breadth was found to increase in areas with high human impact. Stomach analysis of over 400 urban foxes sampled in Zurich, Switzerland revealed a higher proportion of anthropogenic foods such as scavenged meat and pet food (53.6%) compared to natural foods (27.5%) such as rodents, birds, invertebrates and wild fruits (Contesse et al., 2004). In a web-based questionnaire of over 1200 people in Estonia, foxes were found to colonise 33 towns (total surveyed 47; Plumer et al., 2014). Certain behaviours were identified such as feeding on anthropogenic food sources and entering buildings such as greenhouses. However, as urban foxes feed more extensively on anthropogenic food (Contesse et al., 2004; Scholz et al., 2020) and less on natural foods such as rodents and birds that act as paratenic hosts of *Toxocara*, they are likely to be less exposed to infection and hence may harbour fewer *T. canis*.

In the case of the tapeworm *Echinococcus multilocularis* that has some similarities to *Toxocara* in its routes of transmission to definitive hosts, Hegglin et al. (2007) found that plasticity in predation by the fox on the intermediate hosts of *E. multilocularis* influenced potential parasite transmissibility. In this case, the vole, which is a major intermediate host of *E. multilocularis* was less available as a food source for foxes in urban areas and the stomach contents of such urban foxes contained more anthropogenic food items than rodents, hence there was less likelihood of foxes acquiring infection from rodent intermediate hosts. However, in the present meta-analysis, we observed no difference between the pooled prevalence of *Toxocara* in rural versus urban foxes although the number of such studies available for comparison was relatively small (Table 2). In contrast, in one of the most comprehensive studies of the epidemiology of *T. canis* in over 1000 Danish red foxes, the prevalence and abundance of *T. canis* was statistically significantly higher in rural foxes compared to urban foxes (Saeed et al., 2006). More recently, Scholz et al. (2024) found that parasites that undergo trophic transmission were more pronounced in urban Berlin in contrast to rural Brandeburg. The authors concluded that their results did not support the prevailing hypothesis that trophically transmitted helminths are less prevalent in urban areas than in rural areas.

Another factor that may contribute to differences in zoonotic potential between rural and urban foxes is fox density. Fox density is known to vary widely ranging from as little as one fox/km^2^ to as many as 30 foxes/km^2^ in urban areas where food is plentiful (Hoffmann and Sillero-Zubiri, 2016). High fox densities, than can occur in urban areas, are associated with smaller home ranges (Trewhella et al., 1988; Deplazes et al., 2004) that may contribute to particular patterns of environmental contamination. A complex pattern of spatial fox faecal distribution with significantly higher density both on the borders of ploughed fields and on road verges was observed by Guislain et al. (2007) in a rural area of N. E. France, in addition to the marking of territories along roads, pavements and feeding places in private gardens by Giraudoux et al. (2002). Such heterogeneous patterns may undoubtedly contribute to the differential patterns of environmental contamination with *T. canis* eggs and their potential transmissibility to both domestic dogs and paratenic hosts (Otranto and Deplazes, 2019). Clearly, comparison of the potential risk of transmissibility to companion animals and humans in urban versus rural environments requires further attention and may vary considerably depending upon local factors.

As discussed above, the presence of foxes in both urban and rural habitats may result in the release of potentially infective eggs that can be picked up in the environment by domestic dogs, paratenic hosts and humans. However, assessing the level of environmental contamination with such eggs and distinguishing them from those attributable to domestic canids remains challenging. Two interesting approaches to the determination of potential environmental contamination by red foxes involved the collection of fox faecal samples as outlined by Koller et al. (2019) and Gecchele et al. (2020). In Switzerland, Koller et al. (2019) advocated the use of a standardised sampling scheme based on the collection of red fox faecal samples along transects in 1-km^2^ grid cells. The authors concluded that such an approach is a useful procedure for large scale monitoring of, for example, fox density, and could assess the infection pressure on dog populations. A fine scale spatial heterogeneity approach was employed in the urban landscape of Edinburgh (Gecchele et. al., 2020). Over 220 red fox faecal samples were collected and analysed for the presence of helminth eggs and coccidian oocysts. The amount of green space around each sampled site was found to correlate with gastrointestinal parasite prevalence although at the individual species level, *T. canis* eggs did not correlate with any of the chosen variables (such as road cover, traffic counts, population density, greenspace ratio etc). It should be noted that *T. canis* egg density was very low (epg 2.68 in summer and 14.41 in autumn). The number of faecal samples detected was statistically significantly associated with the greenspace ratio and urban wilderness indices.

Utilising a modelling approach, Morgan et al. (2013) utilized a variety of empirical data derived from an urban environment, the city of Bristol, UK. The authors concluded that in the absence of a large population of stray dogs and cats, pet dogs (especially those less than 12 weeks of age), dominate as the major source of total egg output into the environment. However, under certain circumstances, red foxes were found to play a role as contributors to egg contamination. For example, as the rate of removal of dog faeces increased, foxes were predicted to become the main source of *T. canis* eggs. Similar to the findings of Morgan et al. (2013), Nijsse et al. (2015) concluded that Dutch household dogs were found to be the main contributors to environmental contamination with *Toxocara* ova. However, stray cats, owned cats and red foxes also contributed eggs to the environment. The authors concluded that due to the role of stray cats and red foxes (and stray dogs in other contexts), control measures that focus upon household pets alone are not sufficient to reduce environmental contamination to very low levels.

The main strengths of the present study are its comprehensive search, synthesizing data from a wide range of geographic regions and time periods to provide a robust and detailed understanding of the global prevalence of *T. canis* among red foxes. However, like all meta-analyses on proportion studies, our study is also subject to several limitations that should be acknowledged. First, the heterogeneity of the included studies is a significant limitation. Variations in study design, sample size, diagnostic methods and geographic regions can introduce bias and affect the overall estimates of prevalence. While we employed statistical methods to address heterogeneity, it remains a challenge to fully account for these differences. Second, there was a lack or limited data from certain regions (e.g., North Africa, China, Australia). This uneven distribution limits the generalizability of our findings to a global scale and highlights the need for more research in underrepresented areas. Third, the accuracy of diagnostic methods for detecting *T. canis* infection can vary. This could lead to misclassification of infection status and potentially affect the estimated prevalence, affecting the comparability of results across studies. Fourth, the meta-analysis was conducted at the population level, and individual-level data on potential risk factors such as age, sex, and habitat were not available in the majority of studies. This limits the possibility of identifying specific risk factors associated with *T. canis* infection in red foxes. Fifth, the temporal changes in the prevalence of *T. canis* infections among red foxes may not be adequately captured in this meta-analysis. The included studies span several decades, during which changes in environmental conditions, wildlife management practices, and public health interventions could have influenced prevalence rates.

To conclude, based upon the existing data derived from this meta-analysis, the extent of *T. canis* infection in red foxes is considerable with higher pooled prevalences compared to those reported from domestic dogs. However, worm abundances are moderate although such worm burdens may release egg production from density-dependent effects ((Walker et al., 2013)). Further research is needed to address the limitations of this study and provide a more complete global picture of the epidemiology of *T. canis* in red foxes and other wild canids, particularly in underrepresented regions. The global levels of infection that our analysis revealed, coupled with the considerable success and spread of red foxes, particularly in urban environments, enhance their potential as transmitters of *T. canis* to domestic dogs and humans. However, a major challenge remains in understanding the relative contribution of domestic versus wild definitive hosts to environmental contamination with *Toxocara* spp. ova (Holland, 2023). A one health approach that incorporates surveillance and risk assessment of wildlife parasite spillover is clearly required (Waindock et al., 2021).

## CRediT authorship contribution statement

**Celia V. Holland:** Writing – review & editing, Writing – original draft, Methodology, Investigation, Data curation, Conceptualization. **Zahra Geraili Afra:** Methodology, Investigation, Formal analysis. **Soghra Valizadeh:** Investigation, Formal analysis, Data curation. **Maryam Ebrahimi:** Writing – review & editing, Writing – original draft, Formal analysis, Data curation. **Ali Rostami:** Writing – review & editing, Writing – original draft, Software, Methodology, Investigation, Formal analysis, Data curation, Conceptualization.

## Declaration of competing interest

There are no conflicts of interest associated with this manuscript.
