# Supplementary material for: The global prevalence of Toxocara canis among red foxes (Vulpes vulpes): A systematic review and meta-analysis
Source: Int J Parasitol Parasites Wildl. 2024 Sep 4;25:100984. doi: 10.1016/j.ijppaw.2024.100984 (PMC11409046; doi:10.1016/j.ijppaw.2024.100984)
Supplement: Multimedia component 1 [file mmc1.docx]

**Table S1. Main characteristics of eligible studies reporting prevalence of *Toxocara* infection in the red foxes (*Vulpes vulpes*).**

| Author/References | Study duration | Location (within the country) | Country | How collected | Egg count/ worm count | Method (refined) | Sample size | Number of infected | Prevalence |
| --- | --- | --- | --- | --- | --- | --- | --- | --- | --- |
| Northern Europe |  |  |  |  |  |  |  |  |  |
| (Laurimaa et al., 2016) | 2010-2 | Mainland of Estonia | Estonia | Hunted | Worms | Sedimentation & counting technique | 108 | 32 | 29.60% |
| (Tull et al., 2022) | 2019 | Matsalu National Park, Häädemeeste and Hiiumaa | Estonia | Scats collected | Eggs | flotation | 131 | 18 | 13.70% |
| (Willingham et al., 1996) | 1993 | Copenhagan | Denmark | Road kill | Worms | Other | 21 | 17 | 81% |
| (Saeed et al., 2006) | 1997-2002 | 14 Provinces | Denmark | Road kill & hunted | Worms | Other | 1040 | 618 | 59.40% |
| (Al-Sabi et al., 2013) | 2009-12 | Jutland, Mainland & Islands | Denmark | Hunted | Worms | Sedimentation & counting technique | 384 | 234 | 60.90% |
| (Al-Sabi et al., 2014) | 2007 | South Jutland | Denmark | Hunted | Worms | Sedimentation & counting technique | 48 | 31 | 64.6 |
| (Al-Sabi et al., 2014) | 2006-8 | Copenhagan | Denmark | Roadkill | Worms | Intestinal scraping | 70 | 34 | 48.6 |
| (Beresford-Jones, 1961) | 1952-7 | Great Britain | Great Britain |  | Worms | No specific method | 300 | 137 | 45.60% |
| (Williams, 1976) | 1972-74 | Carmarthen & Pembroke counties, S.W. Wales | Great Britain | Ministry of Agriculture | Worms | Intestinal scraping | 149 | 77 | 52% |
| (Hackett and Walters, 1980) | 1973-77 | Wales | Great Britain |  | Worms | No specific method | 280 | 176 | 63% |
| (Richards et al., 1993) | 1986-90 | Bristol | Great Britain | Road kill &other causes | Worms | Other | 521 | 271 | 52% |
| (Richards et al., 1995) | 1985-90 | Salisbury Plain | Great Britain | Hunted & other causes | Worms | Other | 237 | 150 | 63.30% |
| (Richards et al., 1995) | 1985-90 | Windsor Great Park | Great Britain | Hunted & other causes | Worms | Other | 45 | 30 | 66.60% |
| (Richards et al., 1995) | 1985-90 | Bromley | Great Britain | Hunted & other causes | Worms | Other | 40 | 20 | 50% |
| (Smith et al., 2003) | 1999-2000 | Great Britain | Great Britain | Shot | Worms | Intestinal scraping | 588 | 362 | 62% |
| (Wolfe et al., 2001) | 1999-2000 | Dublin & surrounding counties | Ireland | Road kill or shot | Worms | Other | 77 | 29 | 37.7 |
| (Roddie et al., 2008) | 2005 | 6 counties | Ireland | Hunted | Worms | Other | 87 | 53 | 61% |
| (Stuart et al., 2013) |  | Ireland | Ireland | Hunted or road kill, faeces extruded | Eggs | Flotation | 91 | 17 | 19% |
| (Bružinskaitė-Schmidhalter et al., 2012) | 2001-6 | 22 districts, Lithuania | Lithuania | Hunted | Worms | Sedimentation & counting technique | 269 | 109 | 40.50% |
| Western Europe |  |  |  |  |  |  |  |  |  |
| (Suchentrunk and Sattmann, 1994) | 1979-82 | Austria | Austria | Shot or trapped | Worms | Other | 233 | 100 | 42.90% |
| (Losson et al., 1997) | 1993-5 | Province of Luxembourg | Belgium | Shot | Worms | No specific method | 145 | 50 | 34.50% |
| (Brochier et al., 2007) | 2000-4 | Brussels | Belgium | Road or | Worms | Intestinal scraping | 134 | 24 | 18% |
| (Petavy and Deblock, 1980) | 1977-78 | Auvergne | France |  | Worms | No specific method | 69 | 19 | 27.50% |
| (Deblock et al., 1988) | 1984-5 | Massif Central | France | Trapped | Worms | Sedimentation & counting technique | 154 | 79 | 51.30% |
| (Pétavy et al., 1990) | 1983-8 | Haute-Savoie | France | Trapped | Worms | Sedimentation & counting technique | 150 | 67 | 44% |
| (Lamina, 1964) |  | Southern Hessia | Germany |  | Worms | No specific method | 200 | 104 | 52.00% |
| (Loos-Frank and Zeyhle, 1982) | 1976-80 | S.W. Germany | Germany | Shot or road kill or rabies exam | Worms | Other | 3138 | 982 | 31.30% |
| (Lucius et al., 1988) |  | Schleswig-Holstein, N. Germany | Germany |  | Worms | No specific method | 101 | 72 | 71.28% |
| (Ballek et al., 1992) | 1989-90 | N. Hesse & E. Westphalia | Germany |  | Worms | No specific method | 397 | 130 | 32.70% |
| (Wessbecher et al., 1994) | 1989-90 | Karlsruhe | Germany |  | Worms | No specific method | 801 | 242 | 30.20% |
| (Steinbach et al., 1994) | 1991-2 | Siidniedersachsen | Germany |  | Worms | No specific method | 400 | 226 | 56.50% |
| (Pfeiffer et al., 1997) | 1993-4 | Halle & Dessau | Germany |  | Worms | No specific method | 1300 | 344 | 26.50% |
| (Lassnig et al., 1998) | 1993-4 | Styria | Germany | Monitoring | Worms | No specific method | 516 | 242 | 46.80% |
| (Waindok et al., 2021) | 2013-6 | Schleswig-Holstein | Germany | Shot, found dead, trapped, faeces from colon | Eggs | Flotation | 80 | 35 | 43.80% |
| (Scholz et al., 2023) | 2016-8 | Federal states of Berlin & Brandenberg | Germany | Road kill, hunted or natural causes | Worms | Other | 155 | 24 | 15.48% |
| (Borgsteede, 1984) | 1978 | Netherlands | Netherlands | Trapped or shot | Worms | Other | 139 | 101 | 73.70% |
| (Franssen et al., 2014) | 2010-2 | Netherlands border with Germany | Netherlands | Hunted, faeces from colon/rectum | Eggs | Flotation | 136 | 83 | 61% |
| (van den Brakel, 2017) | 2016-7 | Gronigen & Drenthe | Netherlands | Shot | Worms | Intestinal scraping | 69 | 44 | 63.80% |
| (Reperant et al., 2007) | 1998-2002 | Geneva | Switzerland | Road kill | Worms | Sedimentation & counting technique | 228 | 101 | 44.30% |
| (Koller et al., 2019) | 2010-2 | 72 study areas in Switzerland | Switzerland | Scats collected | Eggs | Flotation | 1481 | 179 | 12.10% |
| Southern Europe |  |  |  |  |  |  |  |  |  |
| (Segovia et al., 2004) |  | Andorra | Andorra |  | Worms | No specific method | 53 | 11 | 20.70% |
| (Stevanović et al., 2017) |  | Republic of Srpska | Bosnia & Herzegovina | Faeces | Eggs | No specific method | 21 | 9 | 43.75% |
| (Omeragić et al., 2024) | 2020-2 | Western part of the Balkan Peninsula | Bosnia & Herzegovina | Scats collected | Eggs | Flotation | 313 | 76 | 24.20% |
| (Rajković-Janje et al., 2002) | 1999 | Zagreb County | Croatia | Hunters; rabies control | Worms | Intestinal scraping | 85 | 24 | 28.23% |
| (Papadopoulos et al., 1997) | 1984-6 | Rural areas of Central Greece & 2 islands | Greece | Shot or poisoned | Worms | Intestinal scrapping | 314 | 90 | 28.60% |
| (Soldati et al., 1976) |  | Modenese Apennines | Italy | Rabies monitoring | Worms | No specific method | 23 | 16 | 69.5 |
| (Poglayen et al., 1985) | 1983-4 | Forli Province | Italy | Killed | Worms | No specific method | 103 | 47 | 45.60% |
| (Guberti and Poglayen, 1991) | 1984-7 | Northern Apennines | Italy |  | Worms | No specific method | 153 | 71 | 46.40% |
| (Capelli et al., 2003) | 1990-6 | Vicenza, Pisa, Ferrara Provinces | Italy | Hunted | Worms | No specific method | 109 | 76 | 70% |
| (Capelli et al., 2003) | 1990-6 | Vicenza, Pisa, Ferrara Provinces | Italy | Hunted | Worms | No specific method | 89 | 59 | 66% |
| (Di Cerbo et al., 2008b) | 1998-2006 | Italian Alps | Italy | Natural death or hunted | Worms | Sedimentation & counting technique | 645 | 351 | 54.42% |
| (Di Cerbo et al., 2008a) | 1997-2003 | N.E Italy | Italy | Natural death or hunted | Worms | Sedimentation & counting technique | 260 | 126 | 48.50% |
| (Magi et al., 2009) | 2004-6 | Tuscany | Italy | Hunted | Worms | Sedimentation & counting technique | 129 | 12 | 9.10% |
| (Magi et al., 2016) | 2009-13 | Provinces of Imperia & Cuneo, N.W. Italy | Italy | Culling | Worms | Sedimentation & counting technique | 180 | 48 | 26.70% |
| (Fiocchi et al., 2016) | 2013-4 | Emilia-Romagna region | Italy | Found dead or culling | Worms | Sedimentation & counting technique | 57 | 30 | 52.60% |
| (Varela and Marcos, 1993) | 1970-87 | Portugal | Portugal | Shot | Worms | No specific method | 306 | 34 | 11% |
| (Segovia et al., 2004) |  | Portugal | Portugal |  | Worms | No specific method | 26 | 9 | 34.60% |
| (Eira et al., 2006) | 2000-6 | Portugal | Portugal | Hunted | Worms | No specific method | 62 | 23 | 37.10% |
| (Figueiredo et al., 2016) | 2014-5 | Central West | Portugal | Scats collected | Eggs | Flotation | 28 | 6 | 21.43% |
| (Martins, 2018) |  | Portugal | Portugal | Scats collected | Eggs | Other | 35 | 8 | 22.8%* |
| (Gomes et al., 2023) | 2019-21 | South of the Douro River, C. Portugal | Portugal | Scats collected from transects | Eggs | Other | 44 | 0 | 0% |
| (Alvarez et al., 1995) | 1985-7 | Galicia | Spain | Captured | Worms | Other | 201 | 46 | 23% |
| (Feliu et al., 1996) |  | N.E. Iberian Peninsula | Spain | Road kill or hunted | Worms | No specific method | 105 | 30 | 28.8 |
| (Gortázar et al., 1998) | 1989-93 | Ebro Valley N.E. Spain | Spain | Hunted | Worms | No specific method | 81 | 5 | 6.20% |
| (Criado-Fornelio et al., 2000) | 1997-9 | Guadalajara Province | Spain | Hunted; faeces collected | Eggs | Flotation | 67 | 3 | 4.40% |
| (Segovia et al., 2004) |  | Spain | Spain |  | Worms | No specific method | 132 | 36 | 27.20% |
| (Martínez-Carrasco et al., 2007) | 2001-4 | Murcia, S.E. Spain | Spain | Hunted | Worms | Intestinal scraping | 55 | 25 | 45.50% |
| (Pabón et al., 2010) |  | Catalonia, N.E. Spain | Spain |  | Worms | No specific method | 174 | 21 | 12% |
| (Lledó et al., 2015) |  | Soria Province, S.W. Spain | Spain | Hunted or road kill | Worms | Other | 400 | 10 | 2.50% |
| (Fanelli et al., 2019) | 2006-13 | S.E. Spain | Spain | Surveillance prog. | Worms | No specific method | 287 | 77 | 27% |
| (Figueiredo et al., 2021) |  | Extremadura | Spain | Scats collected | Eggs | Flotation | 21 | 0 | 0 |
| (Arcenillas-Hernández et al., 2024) | 2015-21 | Murcia, S.E. Spain | Spain | Hunted or Road kill | Worms | Intestinal scrapping | 167 | 16 | 9.70% |
| Eastern Europe |  |  |  |  |  |  |  |  |  |
| (Shimalov and Shimalov, 2002) | 1981-2001 | South Belarus | Belarus | Hunted | Worms | No specific method | 94 | 24 | 25.50% |
| (Jancev and Ridjakov, 1977) | 1972-75 |  | Bulgaria |  | Worms | No specific method | 243 | 120 | 49.70% |
| (Kirkova et al., 2011) | 2001-6 | Stara Zagora, S. Bulgaria | Bulgaria | Hunted | Worms | Intestinal scraping | 113 | 24 | 21.40% |
| (Jankovska et al., 2016) | 2010-2 | N. W. Bohemia | Czech Republic | Collected | Worms | Intestinal scraping | 40 | 15 | 37.50% |
| (Széll et al., 2004) |  | Hungary | Hungary |  | Worms | No specific method | 100 | 12 | 12% |
| (Gundlach et al., 1999) |  | Poland | Poland |  | Worms | No specific method | 180 | 28 | 15.55% |
| (Luty, 2001) | 1997-8 | Poznan region | Poland | Hunted | Worms | Other | 92 | 15 | 16% |
| (Pacoń et al., 2006) |  | Lower Silesia | Poland |  | Worms | No specific method | 99 | 28 | 28.30% |
| (Balicka-Ramisz et al., 2003) | 1994-2000 | West region of Poland | Poland |  | Worms | No specific method | 1909 | 657 | 34.40% |
| (Ramisz et al., 2004) | 1997-2000 | S.W. Poland | Poland |  | Worms | No specific method | 380 | 98 | 25.80% |
| (Pilarczyk et al., 2005) | 2004-5 | Western Pomerania | Poland |  | Worms | No specific method | 165 | 56 | 33.90% |
| (Górski et al., 2006) | 2003-4 | Bialowieza Primeval Forest | Poland | Scats collected | Eggs | Flotation | 22 | 3 | 13.60% |
| (Borecka et al., 2009) | 2005-7 | Central Poland | Poland | Hunted | Worms | Intestinal scraping | 639 | 122 | 19.10% |
| (Mizgajska-Wiktor and Jarosz, 2010) | 2007-9 | Wolin Island | Poland | Scats collected | Eggs | Flotation | 22 | 6 | 27.20% |
| (Borecka et al., 2013) | 2011-2 | South Poland | Poland | Scats collected | Eggs | Flotation | 45 | 5 | 11.10% |
| (Tylkowska et al., 2021) | 2008-11 | West Pomerania | Poland | Shot | Worms | Sedimentation & counting technique | 620 | 187 | 30.20% |
| (Gherman et al., 2002) | 1999-2002 | Romania | Romania |  | Worms | No specific method | 50 | 10 | 20% |
| (Barabási et al., 2010) | 2007-10 | 15 counties in Transylvania | Romania | Vet labs, hunted, road kill | Worms | No specific method | 561 | 165 | 29.40% |
| (Ilie et al., 2015) | 2011-2 | Arad County, W. Romania | Romania | Shot | Worms | No specific method | 20 | 0 | 0% |
| (Hora et al., 2016) | 2014-5 | Bihor County, N.W. Romania | Romania | Hunted | Worms | Other | 42 | 15 | 35.71% |
| (Seryodkin et al., 2023) |  | Primorsky Krai , Russian Far East | Russia | Scats collected | Eggs | Flotation | 28 | 1 | 3.60% |
| (Ilić et al., 2016) | 2010-4 | 8 regions of Serbia | Serbia | Shot or hunted | Worms | Other | 172 | 85 | 49.41 |
| (Miljević et al., 2019) | 2015-8 | Vojvodina Province | Serbia | Rabies monitoring | Worms | Intestinal scraping | 223 | 37 | 16.60% |
| (Antolová et al., 2004) | 2002-3 | Slovak Republic | Slovak Republic | Faeces collected from foxes | Eggs | Flotation | 310 | 25 | 8.10% |
| (Letková et al., 2006) | 2000-4 | East Slovak region | Slovak Republic | Hunted | Worms | Intestinal scraping | 302 | 78 | 25.82% |
| (Miterpáková et al., 2009) | 2000-6 | Slovak Republic | Slovak Republic | Hunted; faeces from rectum | Eggs | Flotation | 1198 | 150 | 12.50% |
| (Vergles Rataj et al., 2013) | 2002-5 | Slovenia | Slovenia | Shot | Worms | Intestinal scrapping | 428 | 164 | 38.30% |
| (Pavlović et al., 1997) |  | Belgrade | Yugoslavia | Hunted | Worms | Other | 459 | 231 | 50.32% |
| Central Asia |  |  |  |  |  |  |  |  |  |
| (Ziadinov et al., 2010) | 2006-7 | Atbashy region, Naryn Oblast | Kyrgystan | Hunted | Worms | Sedimentation & counting technique | 151 | 46 | 30.40% |
| (Shakarboev and Berdibaev, 2023) | 2017-22 | Karakalpakstan | Uzbekistan | Hunters & road kill | Worms | No specific method | 62 | 25 | 40.30% |
| North America |  |  |  |  |  |  |  |  |  |
| (Smith, 1978) | 1960 | New Brunswick & Nova Scotia | Canada |  | Worms | Other | 61 | 43 | 70.50% |
| (Wapenaar et al., 2013) | 2004-5 | Prince Edward Island | Canada | Faeces obtained from hunted or trapped | Eggs | Flotation | 271 | 68 | 25.10% |
| (Bouchard et al., 2021) | 2016-7 | Quebec | Canada | Hunted or trapped | Eggs | Flotation | 81 | 15 | 19% |
| (Bouchard et al., 2021) | 2016-7 | Quebec | Canada | Hunted or trapped | Worms | Other | 74 | 2 | 3% |
| (Smith, 1943) | 1938-41 | Iowa | USA |  | Worms | Intestinal scraping | 234 | 21 | 9% |
| (Baron, 1970) | 1969-70 | South Manitoba | USA | Trapped | Worms | No specific method | 75 | 71 | 94.60% |
| (Dyer and Klimstra, 1981) | 1959-64 | South Illinois | USA | Shot or trapped | Worms | Other | 183 | 1 | 0.60% |
| (Dibble et al., 1983) | 1978-81 | Eau Claire, Chippewa, Dunn & Rusk counties, W.C. Wisconsin | USA | Trapped | Worms | No specific method | 78 | 0 | 0 |
| (Kindlin et al., 2013) | 2012 | Franklin County, Pennsylvania | USA | Scats collected | Eggs | Flotation | 40 | 2 | 5% |
| (Brown, 2018) | 2017 | Mount Rainier National Park, South Washington Cascade | USA | Scats collected on trails | Eggs | Flotation | 92 | 12 | 13% |
| Western Pacific |  |  |  |  |  |  |  |  |  |
| (Pullar, 1946) |  | Gippsland district, Melbourne | Australia |  | Worms | No specific method | 67 | 22 | 32.80% |
| (Coman, 1973) | 1968-70 | Victoria | Australia | Shot | Worms | Intestinal scraping | 1320 | 558 | 42.30% |
| (Ryan, 1976) | 1969-74 | New South Wales | Australia | Hunted | Worms | Intestinal scraping | 930 | 327 | 35.20% |
| (Dybing et al., 2013) | 2010 | S.W Australia | Australia | Road kill or culling prog. | Worms | Intestinal scraping | 147 | 22 | 14.90% |
| (Li et al., 2013) | 2010-2 | N. E and S. Qinghai | China | Hunted | Worms | Other | 27 | 0 | 0% |
| (Chen et al., 2022) | 2021 | Shiqu County, Ganzi Tibetan Autonomous Prefecture, Eastern Qinghai-Tibetan Plateau | China | Scats collected | Eggs | Other | 38 | 0 | 0% |
| Eastern Mediterranean & North Africa |  |  |  |  |  |  |  |  |  |
| (Marniche et al., 2018) | 2016 | Réghaia Marsh | Algeria | Scats collected | Eggs | Flotation | 22 | 5 | 22.7%% |
| (Radwan et al., 2009) | 2004 | Giza & Fayoum | Egypt |  | Worms | Other | 41 | 20 | 48.80% |
| (Dalimi et al., 2006) |  | West Iran | Iran | Found dead or shot | Worms | Intestinal scraping | 22 | 1 | 4.54% |
| (Zare-Bidaki et al., 2010) | 2006-8 | Moghan Plain, N.W. Iran | Iran | Found dead or shot | Worms | Sedimentation & counting technique | 89 | 38 | 42.70% |
| (Razmjoo et al., 2014) | 2010-3 | Ilam Province, W. Iran | Iran |  | Worms | Intestinal scraping | 62 | 17 | 27.41% |
| (Kia et al., 2024) | 2021-2 | Zanjan | Iran | Faeces collected from foxes | Eggs | Sedimentation & counting techniques | 30 | 3 |  |
| (Alagaili et al., 2011) |  | Thumamah, Riyadh Province | Saudia Arabia | Faeces collected from rectum; under anaethesia | Eggs | Flotation | 58 | 0 | 0 |
| (Gicik et al., 2009) | 2004-7 | Kars Province | Turkey | Road kill | Worms | Other | 20 | 4 | 20% |
| (GÜRLER et al., 2019) |  | Mid Anatolia & Thrace | Turkey |  | Eggs | Flotation | 409 | 71 | 17.35% |
| (Erol et al., 2021) | 2014-6 | Turkey | Turkey | Hunted | Worms | Sedimentation & counting technique | 103 | 15 | 14.56 |

**Table S2.** Mean abundance, mean intensity and range of *T. canis* worms in red foxes (*Vulpes vulpes*)

| References | Region/Country | Sample size | Mean Abundance | Mean intensity | Range |
| --- | --- | --- | --- | --- | --- |
|  | **Southern Europe** |  |  |  |  |
| (Capelli et al. 2003) | Italy | 89 |  | 6* |  |
| (Capelli et al. 2003) | Italy | 109 |  | 4.3* |  |
| (Capelli et al. 2003) | Italy | 100 |  | 3.5* |  |
| (Di Cerbo et al. 2008) | Italy | 645 | 5.05 | 9.27 | 0-162 |
| (Di Cerbo et al. 2008) | Italy | 260 | 3.64 |  |  |
| (Fiocchi et al. 2016) | Italy | 57 | 3.2 | 6 | 1-30 |
| (Guberti and Poglayen 1991) | Italy | 153 |  | 5.77 |  |
| (Magi et al. 2009) | Italy | 129 | 0.5 | 6.1 | 1-20 |
| (Magi et al. 2016) | Italy | 180 | 1.07 | 4 | 1-37 |
| (Martinez-Carrasco et al. 2007) | Spain | 55 |  | 4.9 | 1-35 |
| (Gortazar et al. 1998) | Spain | 81 |  | 1.4 |  |
| (Feliu et al. 1996) | Spain | 105 |  | 3.9 |  |
| (Fanelli et al. 2019) | Spain | 287 | 0.8 | 7.8 |  |
| (Arcenillas-Hernandez et al. 2024) | Spain | 164 | 0* | 1* | 1-7 |
| (Segovia et. al. 2004) | Spain | 107 | 0.97 |  | 1-20 |
| (Segovia et. al. 2004) | Spain | 25 | 0.80 |  | 1-7 |
| (Eira et al. 2006) | Portugal | 62 |  | 4.61 |  |
| (Segovia et. al. 2004) | Portugal | 26 | 10.35 |  | 1-213 |
| (Segovia et. al. 2004) | Andorra | 53 | 1.96 |  | 1-42 |
| (Rajkovic-Janje et al. 2002) | Croatia | 85 |  | 2.15** | 0-15 |
| (Miljevic et al. 2019) | Serbia | 223 | 0.3 | 1.7 | 1-6 |
|  | **Northern Europe** |  |  |  |  |
| (Al-Sabi et al. 2013) | Denmark | 384 | 2.6 | 4.3 | 1-28 |
| (Saeed et al. 2006) | Denmark | 1040 | 4.1 | 7 | 1-116 |
| (Willingham et al. 1996) | Denmark | 21 | 17.1 |  | 1-55 |
| (Roddie et al. 2008) | Ireland | 87 | 4 |  | 0-54 |
| (Wolfe et al. 2001) | Ireland | 77 | 2.43 |  | 0-32 |
| (Smith 2003) | United Kingdom | 588 |  | 6.1 | 1-103 |
| (Richards et. al. 1995) | United Kingdom | 843 | 4.01 | 7.17 |  |
| (Hackett & Walters 1980) | United Kingdom | 280 | 4.5 |  | 1-29 |
| (Laurimaa et al. 2016) | Estonia | 108 |  | 5 | 1-20 |
|  | **Eastern Europe** |  |  |  |  |
| (Jankovska et al. 2016) | Czech Republic | 40 |  | 3 | 1-24 |
| (Tylkowska et al. 2021) | Poland | 620 |  | 12.2 | 1-84 |
|  | **Eastern Mediterranean & North Africa** |  |  |  |  |
| (Radwan et al. 2009) | Egypt | 41 | 2.3 | 5.4 | 1-17 |
| (Erol et al. 2021) | Turkey | 103 | 0.52 | 3.6 | 1-9 |
|  | **Central Asia** |  |  |  |  |
| (Ziadinov et al. 2010) | Kyrgyzstan | 151 | 2.01 |  |  |
| (Shakarboev and Berdibaev 2023) | Uzbekistan | 62 |  | 20.4 |  |
|  | **Western Pacific** |  |  |  |  |
| (Coman 1973) | Australia | 1320 | 5.9 |  |  |
| (Dybing et al. 2013) | Australia | 147 |  | 7 | 1-34 |
| (Pullar 1946) | Australia | 67 | 4.38 |  |  |
| (Ryan, 1976) | Australia | 930 | 4.3 |  | 0-63 |

* = median abundance or intensity **= geometric mean


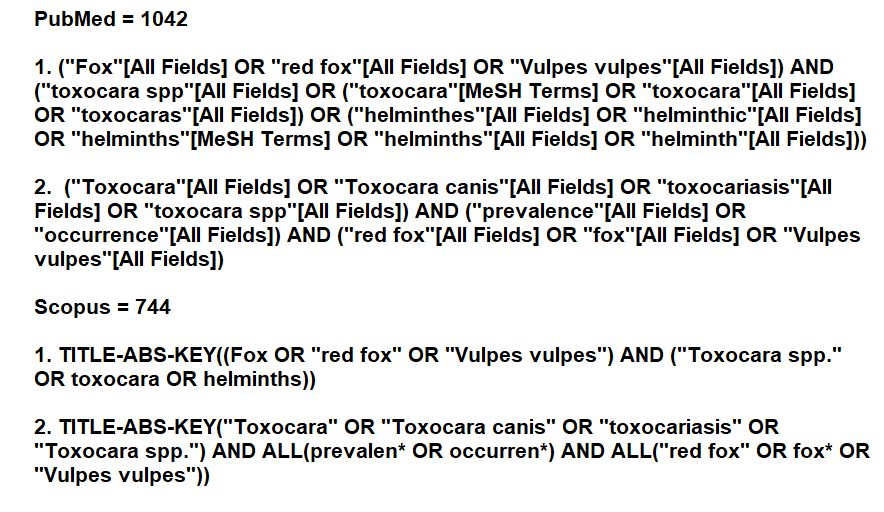


**Figure S1.** Search strategy in PubMed and Scopus


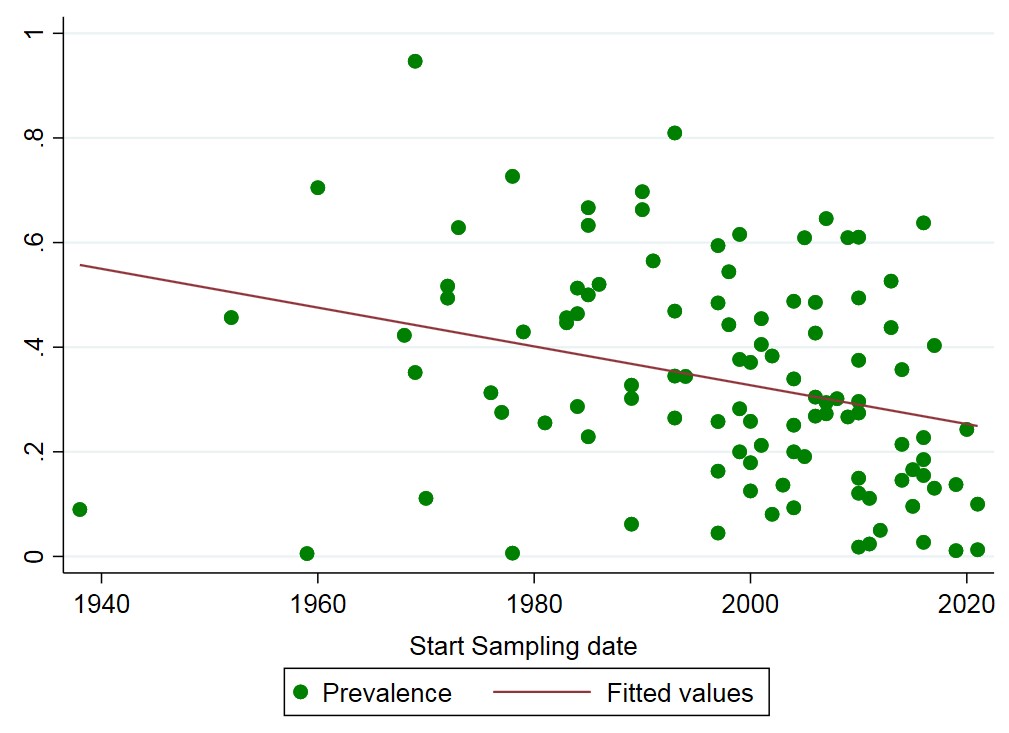


**Figure S2.** Random-effects meta-regression analyses of the prevalence of *Toxocara canis* infection in red foxes according to beginning year of sampling, showing a statistically significant downward trend in prevalence in recent years.


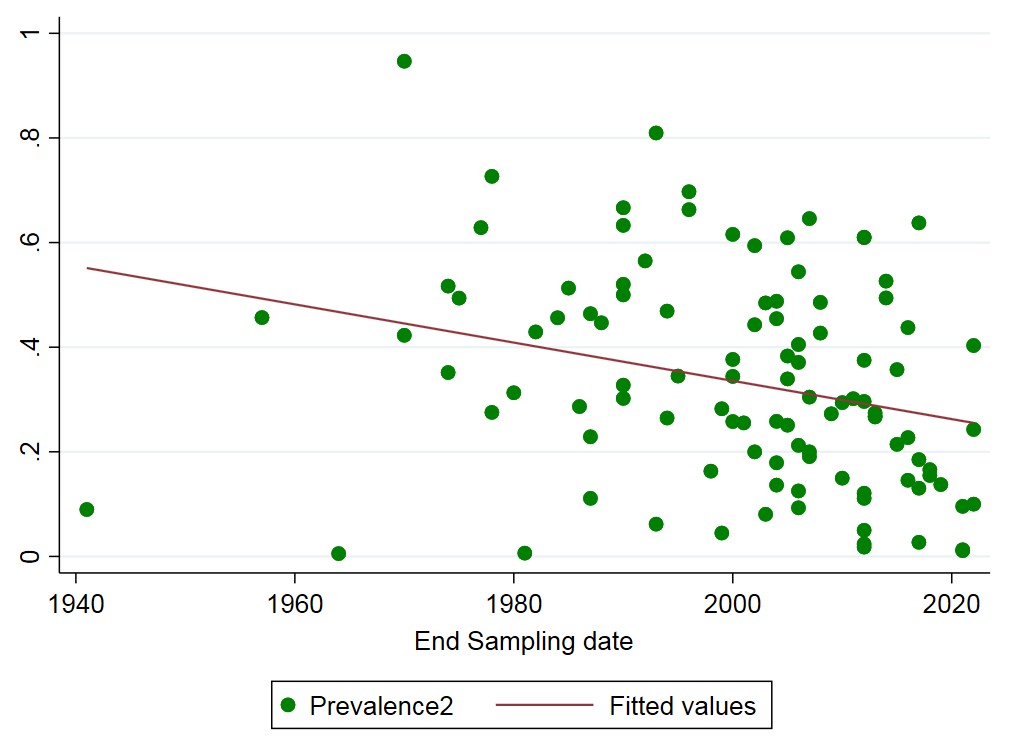


**Figure S3.** Random-effects meta-regression analyses of the prevalence of *Toxocara canis* infection in red foxes according to end year of sampling, showing a statistically significant downward trend in prevalence in recent years.

**References**

Al-Sabi, M.N., Halasa, T., Kapel, C.M., 2014. Infections with cardiopulmonary and intestinal helminths and sarcoptic mange in red foxes from two different localities in Denmark. Acta parasitologica 59, 98-107.

Al-Sabi, M.N.S., Chriél, M., Jensen, T.H., Enemark, H.L., 2013. Endoparasites of the raccoon dog (Nyctereutes procyonoides) and the red fox (Vulpes vulpes) in Denmark 2009–2012–A comparative study. International Journal for Parasitology: Parasites and Wildlife 2, 144-151.

Alagaili, A.N., Mohammed, O.B., Omer, S.A., 2011. Gastrointestinal parasites and their prevalence in the Arabian red fox (Vulpes vulpes arabica) from the Kingdom of Saudi Arabia. Veterinary Parasitology 180, 336-339.

Alvarez, M., Iglesias, R., Garcia, J., Paniagua, E., Sanmartin, M., 1995. Intestinal Helminths of the red fox [Vulpes vulpes L.] in Galicia [Northwest Spain]. Wiadomości Parazytologiczne 41, 429-442.

Antolová, D., Reiterová, K., Miterpáková, M., Stanko, M., Dubinský, P., 2004. Circulation of Toxocara spp. in suburban and rural ecosystems in the Slovak Republic. Veterinary parasitology 126, 317-324.

Arcenillas-Hernández, I., de Ybáñez, M.R., Tizzani, P., Pérez-Cutillas, P., Martínez-Carrasco, C., 2024. Influence of environmental factors on the occurrence of gastrointestinal and cardiopulmonary nematodes in the red fox in the semi-arid Mediterranean areas of the Iberian Peninsula. Research in Veterinary Science, 105199.

Balicka-Ramisz, A., Ramisz, A., Pilarczyk, B., Bieńko, R., 2003. Fauna of gastro-intestinal parasites in red foxes in western Poland. Medycyna Weterynaryjna 59, 922-925.

Ballek, D., Takla, M., Ising-Volmer, S., Stoye, M., 1992. The helminth fauna of the red fox (Vulpes vulpes LINNE, 1758) in Nordhessen and Ostwestfalen. 2. Nematodes. DTW. Deutsche Tierarztliche Wochenschrift 99, 435-437.

Barabási, S.S., Fok, E., Gubányi, A., Mészáros, F., Cozma, V., 2010. Helminth fauna of the small intestine in the European red fox, Vulpes vulpes with notes on the morphological identification of Echinococcus multilocularis. Sci Parasitol 11, 141-151.

Baron, R., 1970. The occurrence of Echinococcus multilocularis Leuckart, 1863 and of other helminths in the red fox, Vulpes vulpes, in southern Manitoba. Canadian Journal of Zoology 48, 1132-1132.

Beresford-Jones, W., 1961. Observations on the helminths of British wild red foxes. Veterinary record 73, 882-883.

Borecka, A., Gawor, J., Malczewska, M., Malczewski, A., 2009. Prevalence of zoonotic helminth parasites of the small intestine in red foxes from central Poland. Med Weter 65, 33-35.

Borecka, A., Gawor, J., Zieba, F., 2013. A survey of intestinal helminths in wild carnivores from the Tatra National Park, southern Poland. Annals of Parasitology 59.

Borgsteede, F., 1984. Helminth parasites of wild foxes (Vulpes vulpes L.) in The Netherlands. Zeitschrift für parasitenkunde 70, 281-285.

Bouchard, É., Schurer, J.M., Kolapo, T., Wagner, B., Massé, A., Locke, S.A., Leighton, P., Jenkins, E.J., 2021. Host and geographic differences in prevalence and diversity of gastrointestinal helminths of foxes (Vulpes vulpes), coyotes (Canis latrans) and wolves (Canis lupus) in Québec, Canada. International Journal for Parasitology: Parasites and Wildlife 16, 126-137.

Brochier, B., De Blander, H., Hanosset, R., Berkvens, D., Losson, B., Saegerman, C., 2007. Echinococcus multilocularis and Toxocara canis in urban red foxes (Vulpes vulpes) in Brussels, Belgium. Preventive veterinary medicine 80, 65-73.

Brown, J., 2018. A parasitological survey of the cascade red fox (Vulpes vulpes cascadensis) and the coyote (Canis latrans) in Mount Rainier National Park. Evergreen State College,

Bružinskaitė-Schmidhalter, R., Šarkūnas, M., Malakauskas, A., Mathis, A., Torgerson, P.R., Deplazes, P., 2012. Helminths of red foxes (Vulpes vulpes) and raccoon dogs (Nyctereutes procyonoides) in Lithuania. Parasitology 139, 120-127.

Capelli, G., Stancampiano, L., Magi, M., Poglayen, G., Guberti, V., 2003. Diversità delle comunità parassitarie intestinali in tre popolazioni di volpi. Journal of Mountain Ecology 7, 199-205.

Chen, Q., Wang, X., Li, C., Wu, W., Zhang, K., Deng, X., Xie, Y., Guan, Y., 2022. Investigation of Parasitic Nematodes detected in the feces of wild carnivores in the Eastern Qinghai-Tibet Plateau, China. Pathogens 11, 1520.

Coman, B., 1973. Helminths parasites of the fox (Vulpes vulpes) in Victoria.

Criado-Fornelio, A., Gutierrez-Garcia, L., Rodriguez-Caabeiro, F., Reus-Garcia, E., Roldan-Soriano, M., Diaz-Sanchez, M., 2000. A parasitological survey of wild red foxes (Vulpes vulpes) from the province of Guadalajara, Spain. Veterinary parasitology 92, 245-251.

Dalimi, A., Sattari, A., Motamedi, G., 2006. A study on intestinal helminthes of dogs, foxes and jackals in the western part of Iran. Veterinary parasitology 142, 129-133.

Deblock, S., Petavy, A., Gilot, B., 1988. Helminthes intestinaux du renard commun (Vulpes vulpes L.) dans le Massif Central (France). Canadian journal of zoology 66, 1562-1569.

Di Cerbo, A., Manfredi, M., Bregoli, M., Milone, N.F., Cova, M., 2008a. Wild carnivores as source of zoonotic helminths in north-eastern Italy. Helminthologia 45, 13-19.

Di Cerbo, A.R., Manfredi, M.T., Trevisiol, K., Bregoli, M., Ferrari, N., Pirinesi, F., Bazzoli, S., 2008b. Intestinal helminth communities of the red fox (Vulpes vulpes L.) in the Italian Alps. Acta Parasitologica 53, 302-311.

Dibble, E.D., Font, W.F., Wittrock, D.D., 1983. Helminths of the red fox, Vulpes vulpes L., in west central Wisconsin. The Journal of Parasitology 69, 1170-1172.

Dybing, N.A., Fleming, P.A., Adams, P.J., 2013. Environmental conditions predict helminth prevalence in red foxes in Western Australia. International Journal for Parasitology: Parasites and Wildlife 2, 165-172.

Dyer, W., Klimstra, W., 1981. Gastrointestinal helminths in red foxes (Vulpes vulpes L) of southern Illinois. Transactions of the Illinois State Academy of Science 74, 137-141.

Eira, C., Vingada, J., Torres, J., Miquel, J., 2006. The helminth community of the red fox, Vulpes vulpes, in Dunas de Mira (Portugal) and its effect on host condition. Wildlife Biology in Practice.

Erol, U., Sarimehmetoglu, O., Utuk, A.E., 2021. Intestinal system helminths of red foxes and molecular characterization Taeniid cestodes. Parasitology Research 120, 2847-2854.

Fanelli, A., Gloria, S.M., Carlos, M.-C., Paolo, T. 2019. Parasites in red-fox: the environmental risk factors (Valencia, Spain). In ESRI Italia.

Feliu, C., Miquel, J., Casanova, J., Torres, J., Segovia, J., Fons, R., Ruiz-Olmo, J., 1996. Helminthfaunas of wild carnivores in the Montseny Massif; an atypical ecosystem in the northeast of the Iberian Peninsula. Vie et Milieu/Life & Environment, 327-332.

Figueiredo, A., Oliveira, L., de Carvalho, L.M., Fonseca, C., Torres, R.T., 2016. Parasite species of the endangered Iberian wolf (Canis lupus signatus) and a sympatric widespread carnivore. International Journal for Parasitology: Parasites and Wildlife 5, 164-167.

Figueiredo, A.M., de Carvalho, L.M., González, M.J., Torres, R.T., Pla, S., Núñez-Arjona, J.C., Rueda, C., Vallverdú-Coll, N., Silvestre, F., Peña, J., 2021. Parasites of the reintroduced Iberian lynx (Lynx pardinus) and sympatric mesocarnivores in Extremadura, Spain. Pathogens 10, 274.

Fiocchi, A., Gustinelli, A., Gelmini, L., Rugna, G., Renzi, M., Fontana, M., Poglayen, G., 2016. Helminth parasites of the red fox Vulpes vulpes (L., 1758) and the wolf Canis lupus italicus Altobello, 1921 in Emilia-Romagna, Italy. Italian Journal of Zoology 83, 503-513.

Franssen, F., Nijsse, R., Mulder, J., Cremers, H., Dam, C., Takumi, K., van der Giessen, J., 2014. Increase in number of helminth species from Dutch red foxes over a 35-year period. Parasites & vectors 7, 1-10.

Gherman, C., Cozma, V., Mircean, V., Brudașcă, F., Rus, N., Detesan, A., 2002. Helminthic zoonoses in wild carnivorous species from Romanian fauna.

Gicik, Y., Kara, M., SARI, B., Kilic, K., MÖ, A., 2009. Intestinal parasites of red foxes (Vulpes vulpes) and their zoonotic importance for humans in Kars province. Kafkas Üniversitesi Veteriner Fakültesi Dergisi 15.

Gomes, F.R., Hipólito, D., Aliácar, S.C., Fonseca, C., Torres, R.T., de Carvalho, L.M., Figueiredo, A.M., 2023. Endoparasites of the Iberian wolf (Canis lupus signatus) and mesocarnivores in Central Portugal. Parasitology Research 122, 435-440.

Górski, P., Zalewski, A., Lakomy, M., 2006. Parasites of carnivorous mammals in Bialowieza Primeval Forest. Wiadomości Parazytologiczne 52.

Gortázar, C., Villafuerte, R., Lucientes, J., Fernández-de-Luco, D., 1998. Habitat related differences in helminth parasites of red foxes in the Ebro valley. Veterinary parasitology 80, 75-81.

Guberti, V., Poglayen, G., 1991. Parasitic zoonoses: survey in foxes (Vulpes vulpes) in the northern Apennines/Zoonosi parassitarie: indagini in volpi (Vulpes vulpes) dell'Appennino settentrionale. Hystrix, the Italian Journal of Mammalogy 3.

Gundlach, J., Sadzikowski, A., Tomczuk, K., 1999. Prevalence of Toxocara canis in foxes and contamination of farm environments with this nematode's eggs. Medycyna Weterynaryjna 55, 255-258.

GÜRLER, A., BÖLÜKBAŞ, C., AÇICI, M., UMUR, Ş., 2019. Determination of Helminths of Red Fox (Vulpes vulpes) by Fecal Examination in Middle Anatolia and Thrace. Kocatepe Veterinary Journal 12.

Hackett, F., Walters, T., 1980. Helminths of the red fox in mid-Wales. Veterinary Parasitology 7, 181-184.

Hora, F., Mederle, N., Ilie, M., Imre, M., Badea, C., Darabuș, G., 2016. The prevalence of gastrointestinal parasites in red foxes (Vulpes vulpes) in Bihor County. Lucrari Stiintifice 49, 67-73.

Ilić, T., Becskei, Z., Petrović, T., Polaček, V., Ristić, B., Milić, S., Stepanović, P., Radisavljević, K., Dimitrijević, S., 2016. Endoparasitic fauna of red foxes (Vulpes vulpes) and golden jackals (Canis aureus) in Serbia. Acta Parasitologica 61, 389-396.

Ilie, M.S., Imre, K., Imre, M., Sorescu, I.D., Hotea, I., Andrei, S., Hora, F., Badea, C., Morariu, S., Dărăbuș, G., 2015. The prevalence of gastrointestinal parasites in red foxes (Vulpes vulpes) from western Romania-preliminary study. Lucrari Stiintifice Medicina Veterinara, 68-73.

Jancev, J., Ridjakov, N., 1977. Helminth fauna of the fox (Vulpes vulpes crucigera Bechstein) in Northwestern Bulgaria. Helminthologia Bulgaria 4, 73-96.

Jankovska, I., Brožová, A., Matějů, Z., Langrova, I., Lukešová, D., Sloup, V., 2016. Parasites with possible zoonotic potential in the small intestines of red foxes () from Northwest Bohemia (CzR). Helminthologia 53, 290-293.

Kia, N.A.T., Haniloo, A., Karamian, M., Torabi, N., 2024. Prevalence of Toxocara canis Infection in Dogs and Foxes in Zanjan, Iran, Using Microscopic and PCR Tests. J Vet Res 79, 9-16.

Kindlin, L.R., Kindlin, C.M., Stewart Jr, R.L., 2013. Survey of the Prevalence and Diversity of Intestinal Parasites Through Scat Analysis of Canids at Letterkenny Army Depot, Franklin County, Pennsylvania. Journal of the Pennsylvania Academy of Science 87, 20-26.

Kirkova, Z., Raychev, E., Georgieva, D., 2011. Studies on feeding habits and parasitological status of red fox, golden jackal, wild cat and stone marten in Sredna Gora, Bulgaria. Journal of Life Sciences 5, 264-270.

Koller, B., Hegglin, D., Schnyder, M., 2019. A grid-cell based fecal sampling scheme reveals: land-use and altitude affect prevalence rates of Angiostrongylus vasorum and other parasites of red foxes (Vulpes vulpes). Parasitology research 118, 2235-2245.

Lamina, J., 1964. Der Parasitenbefall bei Rotfüchsen in Südhessen. Zeitschrift für Jagdwissenschaft 10, 137-142.

Lassnig, H., Prosl, H., Hinterdorfer, F., 1998. Parasites of the red fox (Vulpes vulpes) in Styria. Wiener Tierarztliche Monatsschrift 85.

Laurimaa, L., Moks, E., Soe, E., Valdmann, H., Saarma, U., 2016. Echinococcus multilocularis and other zoonotic parasites in red foxes in Estonia. Parasitology 143, 1450-1458.

Letková, V., Lazar, P., Čurlík, J., Goldová, M., Kočišová, A., Košuthová, L., Mojžišová, J., 2006. The red fox (Vulpes vulpes L.) as a source of zoonoses. Veterinarski arhiv 76, 73-81.

Li, W., Guo, Z., Duo, H., Fu, Y., Peng, M., Shen, X., Tsukada, H., Irie, T., Nasu, T., Horii, Y., 2013. Survey on helminths in the small intestine of wild foxes in Qinghai, China. Journal of Veterinary Medical Science 75, 1329-1333.

Lledó, L., Giménez-Pardo, C., Saz, J.V., Serrano, J.L., 2015. Wild red foxes (Vulpes vulpes) as sentinels of parasitic diseases in the province of Soria, northern Spain. Vector-Borne and Zoonotic Diseases 15, 743-749.

Loos-Frank, B., Zeyhle, E., 1982. The intestinal helminths of the red fox and some other carnivores in southwest Germany. Zeitschrift für Parasitenkunde 67, 99-113.

Losson, B., Mignon, B., Brochier, B., Bauduin, P., Pastoret, P.-P., 1997. Infestation du renard roux (Vulpes vulpes) par Echinococcus multilocularis dans la province de Luxembourg (Belgique): résultats de l'enquête effectuée entre 1993 et 1995. In: Annales de Médecine Vétérinaire.

Lucius, R., Böckeler, W., Pfeiffer, A.S., 1988. Parasiten der haus-, nutz-und wildtiere Schleswig-Holsteins: Parasiten der inneren Organe des Rotfuchses (Vulpes vulpes). Zeitschrift für Jagdwissenschaft 34, 242-255.

Luty, T., 2001. Prevalence of species of Toxocara in dogs, cats and red foxes from the Poznan region, Poland. Journal of Helminthology 75, 153-156.

Magi, M., Guardone, L., Mignone, W., Prati, M., Macchioni, F., 2016. Intestinal helminths of red foxes () in north-west Italy. Helminthologia 53, 31-38.

Magi, M., Macchioni, F., Dell’Omodarme, M., Prati, M., Calderini, P., Gabrielli, S., Iori, A., Cancrini, G., 2009. Endoparasites of red fox (Vulpes vulpes) in central Italy. Journal of wildlife diseases 45, 881-885.

Marniche, F., Milla, A., TIMTAOUCINE, K., BACHA, A., 2018. Coproscopy of wild mammals: the case of red fox Vulpes vulpes (Thomas Say, 1823), common jackal Canis aureus (Linné, 1758) and wild boar Sus scrofa (Linné, 1758) in marsh of Réghaia (Algiers). Oltenia J Stud Natur Sci 34, 89-96.

Martínez-Carrasco, C., Ruiz de Ybáñez, M., Sagarminaga, J., Garijo, M., Moreno, F., Acosta, I., Hernández, S., Alonso, F., 2007. Parasites of the red fox (Vulpes vulpes Linnaeus, 1758) in Murcia, southeast Spain. Rev Med Vet 158, 331-335.

Martins, A.I.P., 2018. A survey of gastrointestinal parasites and trichinella spp. in wild carnivores of Portugal.

Miljević, M., Bjelić Čabrilo, O., Simin, V., Čabrilo, B., Miljević, J.B., Lalošević, D., 2019. Significance of the red fox as a natural reservoir of intestinal zoonoses in Vojvodina, Serbia. Acta Veterinaria Hungarica 67, 561-571.

Miterpáková, M., Hurníková, Z., Antolová, D., Dubinský, P., 2009. Endoparasites of red fox (Vulpes vulpes) in the Slovak Republic with the emphasis on zoonotic species Echinococcus multilocularis and Trichinella spp. Helminthologia 46, 73-79.

Mizgajska-Wiktor, H., Jarosz, W., 2010. Potential risk of zoonotic infections in recreational areas visited by Sus scrofa and Vulpes vulpes. Case study-Wolin Island, Poland. Annals of Parasitology 56.

Omeragić, J., Kapo, N., Škapur, V., Softić, A., Goletić, Š., Šaljić, E., Goletić, T., 2024. Parasites in wildlife in the Federation of Bosnia and Herzegovina. Mac Vet Rev 47, 71-79.

Pabón, M., Mañas, S., Gauss, C., Almería, S., 2010. Coprological examination of endoparasites of red foxes (Vulpes vulpes) from Catalonia, Northeastern Spain. Revista Ibero-Latinoamericana de Parasitología 69, 45-51.

Pacoń, J., Sołtysiak, Z., Nicpoń, J., Janczak, M., 2006. Prevalence of internal helminths in red foxes (Vulpes vulpes) in selected regions of Lower Silesia. Medycyna Weterynaryjna 62, 67-69.

Papadopoulos, H., Himonas, C., Papazahariadou, M., Antoniadou-Sotiriadou, K., 1997. Helminths of foxes and other wild carnivores from rural areas in Greece. Journal of helminthology 71, 227-232.

Pavlović, I., Kulišić, Z., Milutinović, M., 1997. The role of foxes (Vulpes vulpes L.) in the epizootiology and epidemiology of nematode parasitic zoonoses. Acta Veterinaria 47, 177-182.

Petavy, A.-F., Deblock, S., 1980. Helminthes du Renard commun (Vulpes vulpes L.) dans la région du Massif Central (France). Annales de parasitologie Humaine et Comparée 55, 379-391.

Pétavy, A., Deblock, S., Prost, C., 1990. Épidémiologie de l’échinococcose alvéolaire en France I.—Helminthes intestinaux du renard commun (Vulpes vulpes L.) en Haute-Savoie. Annales de parasitologie humaine et comparée 65, 22-27.

Pfeiffer, F., Kuschfeldt, S., Stoye, M., 1997. Helminth fauna of the red fox (Vulpes vulpes LINNE 1758) in south Sachsen-Anhalt--1: Cestodes. DTW. Deutsche tierarztliche Wochenschrift 104, 445-448.

Pilarczyk, B., Balicka-Ramisz, A., Ramisz, A., 2005. The occurrence of intestinal nematodes in red foxes in the Western Pomerania. Wiadomosci Parazytologiczne 51, 249-251.

Poglayen, G., Guberti, V., Leoni, B., 1985. Parasites present in foxes (Vulpes vulpes) of the province of Forli. Parassitologia 27, 303-311.

Pullar, E., 1946. A survey of Victorian canine and vulpine parasites. IV. Nematoda. Australian Veterinary Journal 22, 85-91.

Radwan, N.A., Khalil, A.I., El Mahi, R.A., 2009. Morphology and occurrence of species of Toxocara in wild mammal populations from Egypt. Comparative Parasitology 76, 273-282.

Rajković-Janje, R., Marinculić, A., Bosnić, S., Benić, M., VinkoviĆ, B., Mihaljević, Ž., 2002. Prevalence and seasonal distribution of helminth parasites in red foxes (Vulpes vulpes) from the Zagreb County (Croatia). Zeitschrift für Jagdwissenschaft 48, 151-160.

Ramisz, A., Nicpoń, J., Balicka-Ramisz, A., Pilarczyk, B., Pacoń, J., Piekarska, J., 2004. The prevalence of gastro-intestinal helminths in red foxes (Vulpes vulpes) in the south-west part of Poland. Tierärztliche Umschau 59, 601-604.

Razmjoo, M., Bahrami, A.M., Shamsollahi, M., 2014. Seroepidemiological survey of important parasitic infections of wild carnivores. IJABBR 2, 783-792.

Reperant, L.A., Hegglin, D., Fischer, C., Kohler, L., Weber, J.-M., Deplazes, P., 2007. Influence of urbanization on the epidemiology of intestinal helminths of the red fox (Vulpes vulpes) in Geneva, Switzerland. Parasitology research 101, 605-611.

Richards, D., Harris, S., Lewis, J., 1993. Epidemiology of Toxocara canis in red foxes (Vulpes vulpes) from urban areas of Bristol. Parasitology 107, 167-173.

Richards, D., Harris, S., Lewis, J., 1995. Epidemiological studies on intestinal helminth parasites of rural and urban red foxes (Vulpes vulpes) in the United Kingdom. Veterinary parasitology 59, 39-51.

Roddie, G., Holland, C., Stafford, P., Wolfe, A., 2008. Contamination of fox hair with eggs of Toxocara canis. Journal of helminthology 82, 293-296.

Ryan, G., 1976. Helminth parasites of the fox (Vulpes vulpes) in New South Wales. Australian Veterinary Journal 52, 126-131.

Saeed, I., Maddox-Hyttel, C., Monrad, J., Kapel, C., 2006. Helminths of red foxes (Vulpes vulpes) in Denmark. Veterinary parasitology 139, 168-179.

Scholz, C., Jarquín-Díaz, V., Planillo, A., Radchuk, V., Scherer, C., Schulze, C., Ortmann, S., Kramer-Schadt, S., Heitlinger, E., 2023. Host condition, seasonality and environmental factors explain parasite community differences between urban and rural foxes. Authorea Preprints.

Segovia, J.M., Torres, J., Miquel, J., 2004. Helminth parasites of the red fox (Vulpes vulpes L., 1758) in the Iberian Peninsula: an ecological study. Acta Parasitol 49, 67-79.

Seryodkin, I.V., Kurnosova, O.P., Khrustalev, A.V., Esaulova, N.V., Varlamova, A.I., Odoevskaya, I.М., 2023. Helminth zoonoses of wild carnivore mammals in the Primorsky Krai of the Russian Far East. Российский паразитологический журнал 17, 443-452.

Shakarboev, E., Berdibaev, A., 2023. Ecological and faunistic analysis of helminths of wild mammals from the order carnivora in Karakalpakstan. Adv. Anim. Vet. Sci 11, 1801-1809.

Shimalov, V., Shimalov, V., 2002. Helminth fauna of the red fox (Vulpes vulpes Linnaeus, 1758) in southern Belarus. Parasitology Research 89, 77-78.

Smith, G., Gangadharan, B., Taylor, Z., Laurenson, M., Bradshaw, H., Hide, G., Hughes, J., Dinkel, A., Romig, T., Craig, P., 2003. Prevalence of zoonotic important parasites in the red fox (Vulpes vulpes) in Great Britain. Veterinary parasitology 118, 133-142.

Smith, H., 1978. Parasites of red foxes in New Brunswick and Nova Scotia. Journal of Wildlife Diseases 14, 366-370.

Smith, L.F., 1943. Internal parasites of the red fox in Iowa. The Journal of Wildlife Management 7, 174-178.

Soldati, G., Pavesi, M., Trotti, G., Cocchi, M., Gaiardi, S., Morganti, L., Prosperi, S., Sanguinetti, V., Stanzani, F., 1976. Research on infectious and parasitic agents in foxes of the Modenese Apennines. Wildlife Diseases, 527-528.

Steinbach, G., Welzel, A., v. Keyserlingk, M., Stoye, M., 1994. Zur Helminthenfauna des Rotfuchses (Vulpes vulpes L.) in Südniedersachsen Teil 1: Nematoden und Trematoden. Zeitschrift für Jagdwissenschaft 40, 30-39.

Stevanović, O., Nikolić, S., Mandić, V., Nedić, D., Sekulić, Ž., Pavlović, I., 2017. Toxocarosis and ancylostomatidosis in red foxes (Vulpes vulpes) in Republic of Srpska. Veterinarski Žurnal Republike Srpske 17, 129-136.

Stuart, P., Golden, O., Zintl, A., de Waal, T., Mulcahy, G., McCarthy, E., Lawton, C., 2013. A coprological survey of parasites of wild carnivores in Ireland. Parasitology research 112, 3587-3593.

Suchentrunk, F., Sattmann, H., 1994. Prevalence of intestinal helminths in Austrian red foxes (Vulpes vulpes L.)(Cestoda, Nematoda). Annalen des Naturhistorischen Museums in Wien. Serie B für Botanik und Zoologie, 29-38.

Széll, Z., Sréter, T., Varga, I., 2004. Prevalence, veterinary and public health aspects of gastrointestinal parasites in red foxes (Vulpes vulpes) in Hungary. Magyar Állatorvosok Lapja 126, 293-299.

Tull, A., Valdmann, H., Tammeleht, E., Kaasiku, T., Rannap, R., Saarma, U., 2022. High overlap of zoonotic helminths between wild mammalian predators and rural dogs–an emerging One Health concern? Parasitology 149, 1565-1574.

Tylkowska, A., Pilarczyk, B., Tomza-Marciniak, A., Pilarczyk, R., 2021. The prevalence of intestinal nematodes among red foxes (Vulpes vulpes) in north-western Poland. Acta Veterinaria Scandinavica 63, 19.

van den Brakel, M., 2017. Intensity of Toxocara canis shedding of foxes in the North-Eastern part of the Netherlands.

Varela, M.C., Marcos, M.V., 1993. A helmintofauna da raposa (Vulpes vulpes silacea Miller, 1907) em Portugal. Acta Parasitologica Portuguesa 1, 73-79.

Vergles Rataj, A., Posedi, J., Žele, D., Vengušt, G., 2013. Intestinal parasites of the red fox (Vulpes vulpes) in Slovenia. Acta Veterinaria Hungarica 61, 454-462.

Waindok, P., Raue, K., Grilo, M.L., Siebert, U., Strube, C., 2021. Predators in northern Germany are reservoirs for parasites of One Health concern. Parasitology research 120, 4229-4239.

Wapenaar, W., Barkema, H.W., O’Handley, R., 2013. Fecal shedding of Toxocara canis and other parasites in foxes and coyotes on Prince Edward Island, Canada. Journal of Wildlife Diseases 49, 394-397.

Wessbecher, H., Dalchow, W., Stoye, M., 1994. The helminth fauna of red foxes (Vulpes vulpes linne 1758) in the administrative district of Karlsruhe. 2. Nematodes. DTW. Deutsche tierarztliche Wochenschrift 101, 362-364.

Williams, B., 1976. The intestinal parasites of the red fox in south west Wales. British Veterinary Journal 132, 309-312.

Willingham, A., Ockens, N., Kapel, C., Monrad, J., 1996. A helminthological survey of wild red foxes (Vulpes vulpes) from the metropolitan area of Copenhagen. Journal of helminthology 70, 259-263.

Wolfe, A., Hogan, S., Maguire, D., Fitzpatrick, C., Mulcahy, G., Vaughan, L., Wall, D., Hayden, T., 2001. Red foxes (Vulpes vulpes) in Ireland as hosts for parasites of potential zoonotic and veterinary significance. Veterinary Record 149, 759-763.

Zare-Bidaki, M., Mobedi, I., Ahari, S.S., Habibizadeh, S., Naddaf, S., Siavashi, M., 2010. Prevalence of zoonotic intestinal helminths of canids in Moghan plain, Northwestern Iran. Iranian journal of parasitology 5, 42.

Ziadinov, I., Deplazes, P., Mathis, A., Mutunova, B., Abdykerimov, K., Nurgaziev, R., Torgerson, P., 2010. Frequency distribution of Echinococcus multilocularis and other helminths of foxes in Kyrgyzstan. Veterinary parasitology 171, 286-292.
